# New Heat and Moisture Exchangers for Laryngectomized Patients in Germany: Mixed Methods Study on the Expected Effectiveness

**DOI:** 10.2196/36401

**Published:** 2023-01-11

**Authors:** Anam Ahmed, Janne C Mewes, Iris W A Boot, Hubertus J M Vrijhoef

**Affiliations:** 1 Panaxea Amsterdam Netherlands

**Keywords:** heat and moisture exchanger, HME, laryngectomy, device, laryngeal cancer, mixed methods, review, interview, structured expert elicitation

## Abstract

**Background:**

Notwithstanding the benefits of heat and moisture exchangers (HMEs) in both clinical research and practice, a gap exists between the optimal physiological humidification created through the nasal function and the humidification capacity of HMEs for patients after total laryngectomy. In this study, 5 new HMEs (Provox Life) specialized for situational use with improved humidification capacities were evaluated.

**Objective:**

This study aims to evaluate the effectiveness of the existing HMEs, assess the potential effectiveness of the new HMEs, and elicit expert judgments on the new HMEs’ expected effectiveness and impact on health care use.

**Methods:**

First, a rapid literature review (RLR) was performed to identify evidence on the clinical outcomes, health outcomes, and complications of HMEs in patients who underwent laryngectomy. Second, semistructured interviews with German experts (n=4) were conducted to validate the findings of the RLR and identify reasonable expectations regarding the potential of the new HMEs. Third, a structured expert elicitation among German experts (n=19) was used to generate quantitative evidence on the expected effectiveness of the new HMEs in clinical and health outcomes.

**Results:**

The RLR (n=10) demonstrated that HME use by patients has advantages compared with no HME use concerning breathing resistance, tracheal dryness and irritation, mucus production and plugging, frequency of cough and forced expectorations, sleep quality, voice quality, use of physiotherapy, tracheobronchitis or pneumonia episodes, quality of life, and patient satisfaction. From the expert interviews and structured expert elicitation, it was found that, on average, experts expect that compared with the second-generation HMEs, the new HMEs will lead to a decrease in tracheal dryness or irritation (51%, SD 24%, of patients), mucus plug events (33%, SD 32%, of patients), mucus production (53%, SD 22%, of patients), physiotherapy (0.74, SD 0.70, days) and pulmonary infections (34%, SD 32%) and an increase or improvement in speech quality (25%, SD 23%, of patients), social contacts (13%, SD 18%), quality of life (33%, SD 30%), and patient satisfaction (44%, SD 30%). An improvement in breathing (53%, SD 28%, of patients) and shortness of breath (48%, SD 25%, of patients) was expected. The average number of daily cough periods and forced expectorations was expected to be 2.95 (SD 1.61) and 2.46 (SD 1.42), respectively. Experts expect that, on average, less than half of the patients will experience sleeping problems (48%, SD 22%) and psychosocial problems (24%, SD 20%).

**Conclusions:**

According to German experts, it is expected that the new HMEs with improved humidification levels will lead to additional (clinical) effectiveness on pulmonary health and an improved overall quality of life of patients compared with the currently available HMEs.

## Introduction

### Background

Total laryngectomy (TL) involves the removal of the entire larynx and redirection of the trachea to a neck stoma and is the last surgical treatment option for patients with laryngeal cancer [1,2]. Besides the loss of laryngeal speech, many patients who underwent TL face major pulmonary and other physical and psychosocial postoperative problems, which can negatively affect many health-related quality of life (HRQoL) aspects [3-9]. An important reason for these problems is the disconnection of the upper and lower airways whereby the warming, humidifying, and filtering of inhaled air, before it reaches the pulmonary tract, is no longer possible. Direct inhalation of (more) dry and cold air into the trachea causes pulmonary issues, such as thickening and crusting of the mucus, overproduction of mucosal secretions, and a reduction in the capacity of mucociliary clearance. This leads to an increased need to clear the airway from mucus by forced expectoration, increased involuntary coughing, and shortness of breath [3-5,10]. Moreover, owing to the loss of nasal filtering function and impaired mucociliary clearance, patients who underwent TL have an increased risk of chronic airway inflammation and infections [11-13]. The use of heat and moisture exchangers (HMEs) by patients who underwent TL reduces pulmonary problems. An HME helps by providing stoma occlusion and compensates for the temperature, humidification, and filtering deficit [14-23]. Multiple studies demonstrated the positive effects of HME use on pulmonary rehabilitation and respiratory health in patients, especially when adherence to HME use was high (ie, HME use day and night) [7,15,17,19,21,24-27]. High compliance to HME use has shown positive effects and resulted in decreases in coughing or forced expectorations in patients using HMEs day and night compared with less adherent users [17,28]. In addition, HMEs were found to be cost-effective because of fewer postoperative complications requiring treatments [13,29]. Despite the benefits of HMEs being clear in both clinical research and practice, a gap exists between the optimal physiological humidification level by the nasal function and the humidification capacity of the best HMEs [30]. It is challenging to bridge this gap, as an increase in the humidification capacity of HMEs is constricted by concurrent increases in breathing resistance and HME size. Patient compliance to HME use is also considered an issue, and a large variation in compliance (35%-70%) has been observed [17,28,31]. Many patients experience issues related to the situational use of HMEs, such as the breathing resistance being too high during physical activity, insufficient protection against airborne particles, and pulmonary problems at night [32]. Other reasons for noncompliance are mainly linked to issues related to peristomal adhesives (ie, skin irritation, poor attachment, and poor fit to the stoma shape) [7,28,33]. In this light, new HMEs have been developed focusing on improving HME performance (ie, providing the most optimal humidification possible while keeping the breathing resistance comfortable), situational usability, and HME attachments (improved security, skin-friendliness, and fit). Improvements in these areas will most likely positively influence patients’ HME compliance, pulmonary health, and, ultimately, quality of life (QoL) [32].

### Goal of This Study

In this study, the potential effectiveness of 5 new HMEs (Provox Life) manufactured by Atos Medical AB (Hörby, Sweden) was evaluated. These new HMEs are also called the third-generation HMEs, as they follow the first-generation (Provox HME) and the second-generation (Provox XtraHMEs) HMEs with improved humidification properties compared with the first-generation HMEs. The new third-generation Provox Life HMEs include features that specialize them for situational use (ie, nighttime, home stay, moderate physical activity, high physical activity, and extra protection or filtration) and offer a range of attachments to suit individual patients’ needs. As limited empirical data are available on the use of the new HMEs, information on their expected effectiveness can be collected through expert opinion [34]. An evaluation of the effectiveness of the existing HMEs formed the basis for the assessment of the potential effectiveness of the new HMEs through expert opinion. The objective of this study was 3-fold; we aimed to (1) evaluate the effectiveness of the existing HMEs, (2) assess the potential effectiveness of the new HMEs among German clinical experts, and (3) elicit German expert judgments in a quantitative manner on the new HMEs’ expected effectiveness and impact on health care use.

## Methods

In this mixed methods study, we conducted (1) a rapid literature review (RLR) on the effectiveness of the existing HMEs, (2) interviews with experts to validate the findings of the RLR and identify reasonable expectations regarding the potential of the new HMEs (Provox Life), and (3) a structured expert elicitation (SEE) to elicit experts’ judgments about the new HMEs’ effectiveness and impact on health care use.

### Rapid Literature Review

An RLR, which provides knowledge synthesis in a timelier and resource-efficient manner, was conducted, as empirical data on the new HMEs are limited and expert opinion is essential to assess the potential efficacy of the new HMEs as soon as possible [35,36]. The search was conducted in PubMed. Articles were included if they were clinical studies, randomized controlled trials, reviews, or meta-analyses published between January 2010 and February 2021 in English, Dutch, or German. Articles needed to focus on identifying evidence on the clinical outcomes, health outcomes, and complications of HMEs in patients who underwent TL. Articles were excluded if they were concerning in vitro and ex vivo studies. The search was conducted in February 2021. The search string was as follows: ((laryngectomy AND ((HME OR “Heat and Moisture Exchanger” OR “Hygroscopic Condenser Humidifier”) OR (“Freehands HME”))) OR ((tracheostomy OR tracheotomy) AND (HME OR “Heat and Moisture Exchanger” OR “Hygroscopic Condenser Humidifier”)) OR ((laryngectomy OR tracheostomy OR tracheotomy) AND HMEF). The titles and abstracts were screened independently by 2 authors (AA and JCM). Full papers were retrieved if their titles and abstracts were considered relevant. As for the full-text screening, the selection was done by one author (AA) and checked by a second author (JCM). Disagreements were resolved by discussion, resulting in consensus. Data from each study were extracted using a standardized data abstraction form by one author (AA) and checked by another author (JCM). The extracted data included results on the effectiveness of HMEs in the outcome measures breathing resistance, cough, forced expectoration, tracheal dryness or irritation, mucus production or plugs, speech and voice quality, sleep quality, physiotherapy, pulmonary infection, pneumonia episode, QoL, and patient satisfaction.

### Expert Interviews

Semistructured interviews with experts were conducted to validate the findings of the literature review and identify reasonable expectations regarding the potential of the new HMEs for use in patients who underwent TL. Experts were identified by Atos Medical GmbH (Troisdorf, Germany), as Germany is among the first countries to be introduced to the new HMEs. Experts were approached by the authors by mail. A total of 4 individual interviews were held with medical specialists or senior physicians in the ear-nose-throat field with a focus on oncology and head and neck surgeons at leading institutions in Germany. In this interview, the results of the RLR were discussed for validation, and it was discussed whether the new HMEs with better performance measured in the laboratory would lead to additional effects compared with the currently available HMEs. The following themes were included in the interview: breathing resistance, mucus production or plugs, tracheal dryness or irritation, cough and forced expectorations, sleep quality, speech and voice quality, physiotherapy, pulmonary infections (eg, tracheitis and tracheobronchitis), pneumonia episodes, psychological health, participation in society, QoL, patient satisfaction, and compliance (with HME use). An interview protocol (Multimedia Appendix 1) was prepared, including a brief introduction, an explanation of the research study, the process to be followed during the interview, the main questions, additional follow-up questions, and a final thank you statement. Experts were also introduced to information on the new HMEs (need for development of new HMEs, innovative adaptions made to the new HME devices, the different HMEs included in the new portfolio, and laboratory measurements concerning humidification and breathability of the new HMEs in comparison with their predecessors). The semistructured web-based interviews lasted 60 minutes on average. One independent author (AA) conducted all interviews in March and April 2021, and a second independent author (JCM) took notes during the interviews. Both authors are trained and experienced interviewers. The interviews were videorecorded and transcribed manually. Text segments were assigned a code if they were related to a specific theme, using an inductive, iterative process. The codes were based on the themes included in the interviews. The interviews were analyzed (AA and JCM) based on their themes to gain an insight into the additional effect of the new HMEs (content analysis).

### Structured Expert Elicitation

SEE is a process that allows experts to express their judgment in a statistical and quantitative form. SEE was used to estimate the effectiveness of the new HMEs in health care use. The process consisted of the following steps: selection of experts, characterization of effect measures (ie, effects and use), defining of the scope and format of the elicitation, design of the elicitation protocol, preparation of the elicitation session, elicitation of expert judgments, and data analysis [34]. First, based on the RLR and expert interviews, information on the effect variables of HMEs that are currently being used was collected, which was included in the survey. A survey was set up according to the *bins and chips* method (IWAB and HJMV). In this method, experts were asked to indicate the lower and upper limits of their estimation of a specific effect variable (eg, difficulties with breathing) [37]. Experts assigned weights (0-100) to the effect variable to indicate the weight of their effect estimate in different intervals (0-10, 11-20, 21-30, etc). The stronger the expert believed that a specific effect lies in a given interval, the greater the weight for that interval was. This provides an estimated effect distribution (a mean effect and spread). For every expert judgment on each effect variable, a mean was calculated. Subsequently, the sample mean was calculated, which indicated the estimation of all expert judgments combined. For the analysis, we multiplied the weight by the average of the intervals. Thereafter, the sum of these outcomes was divided by the total weights assigned by the experts. In this manner, a weighted average per expert was established. The sum of all weighted averages of experts was averaged, and the corresponding SDs were calculated, resulting in our final indicator of experts’ beliefs. For some effect variables, the experts were asked to indicate the lower and upper limits of their estimation in absolute numbers. For these effect variables, the last category would not be a set interval (eg, 60-70), but it ended with a category “equal or more than X (i.e., ≥X).” In cases where an expert could be pointing at an amount higher than X, the assigned weight was multiplied by X + 1 interval. An example of a question with fictional answers is provided in Multimedia Appendix 2. The participants were sent the electronic survey contained in Multimedia Appendix 3 (Atos Medical, unpublished data, 2021, [12,16,28,29,31,38-44]) via a web link (SurveyMonkey, Momentive Inc). German experts were identified by Atos Medical GmbH and approached by an independent author (IWAB) via mail, including the 4 experts interviewed in the previous step of this study. Before participating in the web-based survey, the experts were required to read a white paper on the new HMEs and their developmental background and watch a video presentation about the new HMEs’ product portfolio to provide information on the properties of the new HMEs. If the experts did not read the white paper and watch the video presentation and were, therefore, assumed to have no basic knowledge of the new HMEs, they could not take part in the survey. The survey started with 6 general questions regarding current profession, years of experience in caring for patients with laryngeal cancer, years of experience in caring for patients who underwent TL, years of experience in prescribing and/or monitoring the use of HMEs, years of experience as a scientific researcher in the field of laryngeal cancer, and whether they had prescribed the new HMEs. The experts were also asked whether they had read the white paper on the new HMEs and watched the video on the new HMEs. The survey contained 16 other questions about the following effect variables: breathing, shortness of breath, tracheal climate (n=2), mucus production or plugging (n=2), coughing, forced expectorations, sleep quality, speech quality, psychological aspects, physiotherapy, tracheobronchitis or pneumonia episodes, social contacts, QoL, and patient satisfaction. At the end of the questionnaire, the participants could provide general comments or remarks. The participants were given 1 week to complete the questionnaire. After 1 week, a reminder email was sent to complete the questionnaire within a week. The analysis was conducted by 1 author (IWAB) and checked by 2 other authors (AA and HJMV).

### Ethics Approval

As this study did not involve patients or study participants, an ethical research approval was not needed according to Article 1b of the Dutch Medical Research in Human Subjects Act [45]. Notwithstanding, all the participants provided their consent, and all the data were processed anonymously. The participants could withdraw from the study at any time without any consequences. The participants were financially compensated for their input in the SEE questionnaire.

## Results

### RLR Results

A total of 62 publications were identified in PubMed. After screening the titles and abstracts of these studies, the full text of 38 (61%) of them were assessed. Of these 38 studies, 10 (16%) were included in the analysis. In total, 3 (30%) randomized controlled trials [28,29,40], 3 (30%) time-series studies in which patients acted as their own controls [31,41,42], 1 (10%) retrospective comparative cohort study [43], 1 (10%) retrospective clinical and survey study [12], 1 (10%) multigroup design study (study design was not reported by the authors themselves) [16], and 1 (10%) case-control study [44] were included. The new generation of HMEs were compared with a control (no HME use) [12,16,28,39-41], an external humidifier [29,43,44], or a previous generation HME [31]. The studies were performed in 7 different countries, namely the United States [16,43], Canada [44], France [29,40], Italy [41,42], Spain [31], the Netherlands [12], and Poland [28]. The studies included a total of 550 patients, with a minimum of 30 patients [41,42] and a maximum of 89 patients [12].

### Effects of HMEs

In Table 1, the effects reported by these studies are shown. Breathing was equal or less strenuous for HME users when compared with the control group [41]. A statistically nonsignificant effect with regard to breathing effort was achieved in 1 (10%) study [40]. Patients also reported a statistically significant decrease in shortness of breath [28,42]. However, 1 (10%) study reported a higher shortness of breath [16]. No difference was experienced in breathing resistance compared with a previous generation HME device [31]. Moreover, improvements in the tracheal climate were observed, as patients experienced significantly less tracheal dryness or irritation when using an HME [41,42] or when using an HME with improved humidification capacities [31]. Studies also reported statistically significantly lower mucus production and a lower rate of mucus plugging in patients who used an HME [42-44]. A statistically nonsignificant improvement was observed in mucus production compared with a control [41] or when using an HME with better humidification capacities [31]. Statistically significant effects of HME use also included a significant decrease in coughing (episodes) [28,29,40,42] and daily forced expectorations [28,29,42]; 2 (20%) studies reported a statistically nonsignificant difference in the number of coughs or forced expectorations when compared with a previous generation HME [31] or a control [41]. Moreover, HME use has been linked to statistically significant improvements in sleep, as patients experience fewer sleep disturbances [29,42]. However, 4 (40%) studies reported no statistically significant difference in sleep quality [16,28,31,44]. A statistically significant improvement in speech quality was observed in HME-compliant patients [42]. A statistically nonsignificant improvement in voice quality was found in patients using HME [16,28,31,40]. One (10%) of the studies reported a statistically significant difference in psychological stress [42], whereas in the other studies, patients reported no differences in psychosocial aspects [16,28,31]. HME use also significantly reduced the number of days requiring physiotherapy after surgery [44]. The number of tracheobronchitis or pneumonia episodes per patient per year in non-HME users was statistically significantly higher than that in HME users [12]. When using a hands-free device, patients have statistically significantly more frequent social contacts [16]. One (10%) of the studies reported a statistically nonsignificant improvement in social contacts [42]. The QoL of patients who underwent laryngectomy statistically significantly increased in 1 (10%) study [42] but not in the other study that examined this [40]. A statistically significant increase in patient satisfaction was observed in 1 (10%) study [29], and a statistically nonsignificant difference was observed in 2 (20%) studies [41,42].

**Table 1:** Overview of the effects of heat and moisture exchangers (HMEs) as reported in the included studies.

Effect variables and results^a^		References
**Breathing**		
	After 12 weeks of using an HME, only 1 patient (3.4%) found it more difficult to breathe through the HME, 7 patients (24.1%) felt no difference, and 21 (72.4%) patients found breathing through the HME less difficult (*P*=.002).	Macri et al [41], 2016
	At 6 weeks, 71% of the patients and at 3 months, 88% of the patients responded positively to the question “Are you breathing better?”.	Dassonville et al [40], 2011
**Shortness of breath**		
	A statistically significant decrease in shortness of breath was demonstrated with a baseline value of 5.7 and a value of 3.8 after 6 and 12 weeks of Provox XtraHME (*P*<.0001) using structured questionnaires.	Parrilla et al [42], 2015
	Both the HME users and the no-HME control group experienced a decrease in shortness of breath at rest (HME group: *P*=.03; control group: *P*=.006). The HME users also experienced a significant increase in shortness of breath while climbing steps (*P*=.012).	Bień et al [28], 2010
	In total, 35% of the patients experienced lower breathing resistance with the first-generation HMEs, 25% experienced lower breathing resistance with the second-generation HMEs, and 40% experienced no difference between the 2 devices (*P*=.41).	Herranz et al [31], 2013
	Non-HME users, Provox Micron HMEs users (first generation), and Provox HME (first generation) users scored a 4.6, 4.9, and 4.3, respectively, on the Quality-of-Life Questionnaire (*P*=.363).	Brook et al [16], 2013
	After 12 weeks of Provox XtraHME (second generation) use, 3.4% of the patients found it more difficult to breathe through the HME, 24.1% of the patients felt no difference, and 72.4% of the patients found breathing through the HME less difficult (*P*=.002).	Macri et al [41], 2016
**Tracheal climate**		
	After 2 weeks of Provox XtraHME (second generation) use, 60% of the patients reported less tracheal dryness or irritation and 40% reported no changes, compared with no HME use at baseline. After 12 weeks, 82.8% of the patients reported less irritation, 13.8% reported no changes, and 3.4% reported more irritation (*P*=.013).	Parrilla et al [42], 2015
	After 12 weeks, 82.8% of the HME-using patients reported less irritation, 13.8% reported no changes, and 3.4% reported more irritation (*P*=.013).	Macri et al [41], 2016
	Patients reported significantly less tracheal dryness with the second-generation HMEs (38%) than with the first-generation HMEs (14%) (*P*=.039). Moreover, 42.5% of the patients preferred the second-generation HMEs, 40% preferred the first-generation HMEs, and 17.5% had no preference.	Herranz et al [31], 2013
**Mucus production or plugging**		
	The rate of mucus plugging was significantly lower in the XtraHME group than in the EH^b^ group (0.13 and 0.38 per 10 inpatient days, respectively, *P*=.02). The proportion of patients with ≥1 mucus plug events was also significantly reduced in the HME group (50% in the EH group and 11% in the HME group, *P*=.01) in the postoperative period.	Ebersole et al [43], 2020
	After 2 weeks of Provox XtraHME (second generation) use, there was statistically significantly less mucus production in patients.	Parrilla et al [42], 2015
	Of those experiencing mucus plugging, 12.5% had used an HME, in contrast to 87.5% who used an EH. There was a significant difference between case and control groups based on use of an HME (χ^2^=9.4, *P*=.002). The odds ratio of a mucus plug event when not using HME was 8.27.	Foreman et al [44], 2016
	After 12 weeks of use, 36% of the patients reported less mucus production with the first-generation HME, 26% reported less mucus production with the second-generation HME, and 41% reported no difference (*P*=.162).	Herranz et al [31], 2013
	After 12 weeks of Provox XtraHME use, 79.3% of the patients reported less mucus production, 6.9% reported more mucus production, and 13.8% reported the same mucus production (*P*=.368).	Macri et al [41], 2016
**Coughing**		
	At baseline (control) and after 2, 6, and 12 weeks of XtraHME use, the average number of daily coughs was 8.8, 4.6, 3.5, and 2.4, respectively (*P*<.001). After 2 weeks of HME use, the patients reported less coughing (63.3%) compared with the baseline phase. After 6 and 12 weeks of HME use, the patients did not report any further change in coughing (*P*=.337).	Parrilla et al [42], 2015
	At 3 months, there was a significant decrease in coughing in the HME group versus the no-HME control group (*P*=.00174). By means of an analog scale ranging from 0 to 10, the baseline value was 4, and after 2 months, this value dropped to 2.	Dassonville et al [40], 2011
	A decrease was observed in the frequency of coughing in the HME group (*P*<.001). In the control group, the frequency of coughing in week 1 was 60 times and in week 12, 56 times. In the HME group, this was 48 and 30, respectively.	Bień et al [28], 2010
	The number of coughing episodes were significantly lower in the HME arm (*P*<.001). In the EH group, 73% of the patients had 2 to 10 spontaneous coughing episodes per day, whereas 8% had 20, another 8% had 30, and 4% had 72 episodes a day (for 8%, this information was missing). In the HME group, most patients (90%) had 1 to 5 spontaneous coughing episodes per day, whereas 4.3% had 10 and another 4.3% had 20 such episodes per day.	Mérol et al [29], 2012
	After 2 weeks of HME use, 63.3% of the patients reported less coughing compared with the baseline period. After 6 and 12 weeks of HME use, 83.3% and 89.7% of the patients, respectively, did not reported any further change in coughing (*P*=.337).	Macri et al [41], 2016
	The combined number of coughs and forced expectorations using the first- and second-generation HMEs was similar (*P*=.304). The average daily coughing frequency was lower when using the second-generation HMEs (2.0 vs 2.59 per day). Most patients (51%) felt no difference between the 2 HMEs (*P*=.275) or reported that the second-generation HMEs performed better (35% vs 22%).	Herranz et al [31], 2013
**Forced expectorations**		
	A significant decrease in forced expectoration was reported in the XtraHME (second generation) group versus the control group (*P*<.0001). At baseline (control) and after 2, 6, and 12 weeks of Provox XtraHME use, the average number of daily forced expectorations was 6.3, 3.0, 2.3, and 1.9, respectively.	Parrilla et al [42], 2015
	A decrease in the frequency of forced expectorations (*P*<.001) was found in the HME group compared with the control group. In the control group, the frequency of forced expectorations in week 1 was 59 and in week 12, 53. In the HME group, this was 56 and 27, respectively.	Bień et al [28], 2010
	The frequency of mucus expectoration for clearing the trachea was significantly lower in the HME arm (*P*<.001). In the EH group, the mean frequency of mucus expectoration was 5.5 times per day and in the HME group (first generation), 2.5 times per day.	Mérol et al [29], 2012
	The combined number of coughs and forced expectorations using the first- and second-generation HMEs was similar (*P*=.304 and *P*=.764, respectively). The maximum number of forced expectorations was lower using the second-generation HME (12 vs 20 times per day). In total, 47% experienced no difference between the 2 generation HMEs, 23% experienced less forced expectoration with the first-generation HMEs, and 30% experienced less forced expectoration with the second-generation HMEs (*P*=.513).	Herranz et al [31], 2013
**Sleep quality**		
	A statistically significant improvement in sleep quality with a baseline value of 7.1 and a value of 6.2 (a lower score indicates less burden for the patient) after 12 weeks of Provox XtraHME use (*P*=.004) was reported in the structured questionnaires.	Parrilla et al [42], 2015
	Sleeping disturbances were significantly lower in the HME group (*P*<.001): 83% of patients in the HME group arm did not experience any sleeping discomfort over the hospitalization period, whereas 17% mentioned some sleeping discomfort.	Mérol et al [29], 2012
	No significant difference was reported between the HME group and EH group in sleep quality.	Foreman et al [44], 2016
	Patients did not report a difference in sleeping when using the first- or second-generation HME (72%).	Herranz et al [31], 2013
	In the control group, almost all the patients (97.5%) had sleeping problems, and this did not change over time. In the full-compliance HME group (first generation), 79% of the patients had sleeping problems at baseline, and 72% had this problem after 3 months of HME use. This reduction was not significant.	Bień et al [28], 2010
	Non-HME users, Provox Micron HMEs users, and Provox HME users scored a 4.5, 4.8, and 4.6, respectively, on the Quality-of-Life Questionnaire (*P*=.913).	Brook et al [16], 2013
**Speech quality**		
	A statistically significant improvement in speech quality, with a baseline value of 12.3 versus 10.3 after 12 weeks of Provox XtraHME (*P*<.0001) use, was reported in the structured questionnaires (a lower score indicates a lower burden for the patient).	Parrilla et al [42], 2015
	At 6 weeks, 80% of the patients experienced a statistically nonsignificant improvement in stoma occlusion with more powerful and more audible phonation. At 3 months, this number rose to 94%. At 6 weeks, 52% of the patients using a voice implant and an HME showed an improvement in the intensity of the prosthetic voice, and at 3 months, this value increased to 71% (>5 on the analog scale). Regarding the fluency of speech, 62% of the patients experienced a nonsignificant improvement after 6 weeks of HME use, which increased to 76% after 3 months. Moreover, 71% of the patients had perceived vocal improvement at 6 weeks, and 81% had perceived vocal improvement at 3 months.	Dassonville et al [40], 2011
	The HME users and Micron users reported a better voice than did the non-HME users (not statistically significant). Non-HME users, Provox Micron HMEs users, and Provox HME users scored a 7.6, 9.4, and 8.0, respectively, on the Quality-of-Life Questionnaire (*P*=.396).	Brook et al [16], 2013
	No significant change was reported in voice and speech aspects over time in the control and HME group. A trend was seen for the prosthetic speakers to report more fluent speech with HME use (*P*=.073).	Bień et al [28], 2010
	After 6 weeks of use, no difference was reported in speech intelligibility and voice (72%, *P=*.739) between Provox HME (first generation) and Provox XtraHME (second generation) users.	Herranz et al [31], 2013
**Psychosocial aspects**		
	Patients reported no differences concerning psychosocial aspects. Among both patients using the first-generation HMEs and those using the second-generation HMEs, over 75% reported to have no problems, socially or psychologically.	Herranz et al [31], 2013
	At baseline, most patients (80%-90%) reported no or only slight problems with anxiety and depression, which had significantly increased in the control group (no HME use; *P*=.003); however, in the HME groups (first generation), no significant changes were found.	Bień et al [28], 2010
	A statistically significant improvement in psychological stress, with a baseline value of 7.1 versus a value of 5.1 after 12 weeks of Provox XtraHME use (*P*<.001), was reported in the structured questionnaires (a lower score means less burden for the patient).	Parrilla et al [42], 2015
	Non-HME users, Provox Micron HMEs users, and Provox HME users scored a 5.8, 6.5, and 6.6, respectively, on the Quality-of-Life Questionnaire (*P*=.688).	Brook et al [16], 2013
**Physiotherapy**		
	The use of Provox XtraHME significantly reduced the number of days requiring chest physiotherapy after surgery (1.75 vs 3.20 days, *P*=.034) compared with the use of EH.	Foreman et al [44], 2016
**Tracheobronchitis or pneumonia episodes**		
	Among the non-HME users, 0.285 tracheobronchitis or pneumonia episodes was reported per patient per year, which was statistically higher than the 0.066 episodes reported per patient per year among the HME users (first generation; *P*=.047). Among the non-HME users, an average of 0.129 pulmonary infections (tracheobronchitis and pneumonia together) was documented per patient per year. Among the HME users (first generation), this average was 0.092 per patient per year (*P*=.33).	van den Boer et al [12], 2014
**Social contacts**		
	Patients who use a Provox FreeHands device tended to have more frequent social contacts (*r*=0.251; *P*=.030).	Brook et al [16], 2013
	A statistically nonsignificant improvement in social contacts, with a baseline value of 8.1 versus a value 8.3 after 12 weeks (*P*=.728), was reported in the structured questionnaires when comparing no HME use with HME (second generation) use.	Parrilla et al [42], 2015
	Non-HME users, Provox Micron HME (first generation) users, and Provox HME (first generation) users scored a 9.6, 8.4, and 9.7, respectively, on the frequency of social contacts category from the Quality-of-Life Questionnaire (*P*=.438; a higher score indicates more problems).	Brook et al [16], 2013
**QoL^c^**		
	The EQ-5D Index showed an increase throughout the study, with an increase from an average of 0.84 at baseline to 0.96 after 12 weeks of HME use (*P*<.001). The EQ-5D VAS^d^ scale showed an increase from 61.3 at baseline to 80.0 after 12 weeks of Provox XtraHME use (*P*<.0001).	Parrilla et al [42], 2015
	Among 60 patients, who were randomized between a control group that used no device and a group equipped with the Provox HME (first generation), 92% of the patients with the device perceived an improvement in their QoL at the end of 3 months (>5 on the analog scale).	Dassonville et al [40], 2011
**Patient satisfaction**		
	Patients’ satisfaction showed a significant improvement of first-generation HME over EH (*P*<.001). Patient satisfaction with the EH was quite low: 11% of the patients reported that they were satisfied with it, 8% reported they somewhat liked it, and 81% reported that they did not like it. All the patients (100%) in the HME (first generation) group were satisfied with the device.	Mérol et al [29], 2012
	After 12 weeks of use, 60.7% of the patients were “very satisfied” with the use of the HME (first generation), and 39.3% were “satisfied.” None of the patients was dissatisfied with XtraHME (second generation).	Macri et al [41], 2016

^a^A *P* value is presented if reported in the paper.

^b^EH: external humidifier.

^c^QoL: quality of life.

^d^VAS: visual analog scale.

### Expert Interviews

All 4 experts stated that they would expect the use of the new HMEs to lead to improved outcomes compared with the use of the current HMEs. Because the optimized breathing resistance adapted to different situations while also maintaining high humidification levels by the HMEs of the new portfolio, it is likely that patients will tend to use the HME more often. One of the experts stated that “a better product that has significant benefits for the patient would lead to a higher compliance (participant 1).” All 4 experts were convinced that improved humidification, breathability, and situational usability together with improved HME attachments will lead to an optimized breathing experience with limited shortness of breath. One of the experts interviewed said that “if you change some problems in current available HMEs like humidity and breathing resistance, probably the compliance will increase (participant 2).” All the experts pointed out that with a better performance on the humidification and breathing resistance ratio, the tracheal climate would improve, as this leads to higher humidification and improved temperature. Experts indicated that they expect that when using the new HME, patients will experience less irritation when inhaling cold or dry air or small particles. A better HME with increased humidification levels will also reduce excess mucus and mucus plugs, (involuntary) coughing, and forced expectorations. Consequently, the sleep quality will get improved. The new HMEs would prevent patients from having dry and hard sticky mucus, which often needs to be removed mechanically with a forceps. Moreover, complications such as a reduced diameter of the trachea due to mucus obstruction affecting the trachea’s functionality were expected to occur less frequently. By preventing these complications, patients are expected to experience breathing problems less often. There was a consensus among experts that because of the larger positive effects of the new HMEs compared with current HMEs and higher adherence to HME use, the rates of inflammation and infection (eg, tracheitis, tracheobronchitis, and pneumonia episodes) will decrease, resulting in reduced care consumption by the patient (eg, fewer hospital readmissions) and, thus, reduced health care costs. One of the experts mentioned that the “length of hospital stay, if even it happened, will be less, because people get better and hospital readmission will probably even decrease (participant 1)”. An overall improvement in pulmonary health because of the use of the new HMEs will lead to an increase in the HRQoL and satisfaction of the patient. In turn, this will lead to an improved psychological health in the patient. Patients who can cope well with coughing and other symptoms feel confident to participate in social activities. Improvements in all these domains will contribute to a higher participation of the patient in society, and for patients of working age, these increase the chances of returning to work. This would entail patients having more social contact. Even though one of the experts mentioned that “voice quality is one of the most important things for patients with laryngectomy, (participant 3)” overall, the experts did not expect to see any effects of using the new HMEs on speech, voice quality, or the requirement of physiotherapy.

**Figure 1:**
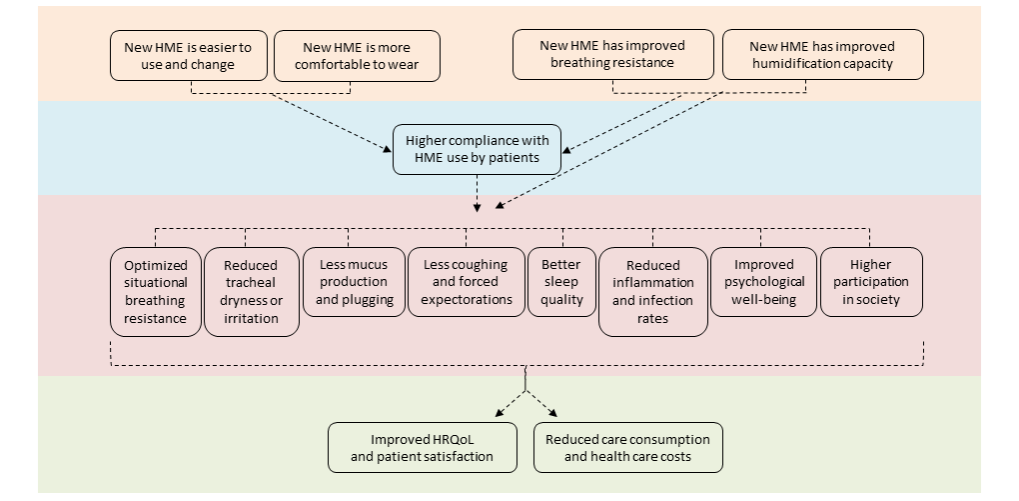
Expected outcomes of the new improved heat and moisture exchangers (HMEs) according to the experts. HRQoL: health-related quality of life.

In Figure 1, the statements in the upper boxes (orange part) are based on laboratory measurements, patient-reported outcomes, and user evaluations of the new-generation HMEs, which result in the box in the blue part. The boxes in the pink layer contain the effects of using HMEs, which the experts believe will further improve when using the new-generation HMEs, resulting in the outcomes given in the boxes in the green part. The causal relationships among the effects in the pink section were not explicitly discussed with the experts; however, they indicated that the effects were all related to each other.

### Structured Expert Elicitation

A total of 24 German experts were invited to participate in a web-based survey. Of those invited, 21 (88%) experts agreed to participate, of whom 19 (90%) completed the survey. These participants were head and neck surgeons (n=11, 58%), otolaryngologists (n=3, 16%), and speech therapists (n=5, 26%). A total of 15 (79%) experts had heard about the new HMEs before but had never prescribed them, whereas 4 (21%) experts had prescribed the new HMEs at least once. Experts had an average of 18.00 (SD 7.86) years of experience with providing care to patients with laryngeal cancer, 17.32 (SD 8.09) years with providing care to patients who underwent TL, 13.05 (SD 5.85) years with prescribing and/or monitoring the use of HMEs, and 7.95 (SD 9.06) years as a scientific researcher in the field of laryngeal cancer.

The average expert judgments are listed in Table 2 (refer to Multimedia Appendix 4 for the average scores per interval).

Experts believed that, on average, 51% (SD 24%) of patients will experience a decrease in tracheal dryness or irritation when comparing the use of the new HMEs to a previous generation HME after 12 weeks of use. An average decrease of 33% (SD 32%) was expected in patients experiencing mucus plug events when using the new HMEs compared with patients using second-generation HMEs in the postoperative period. It was expected that after 12 weeks of using the new HMEs, 53% (SD 22%) of patients will have decreased mucus production compared with no HME use. On average, 25% (SD 23%) of patients were expected to have better speech quality after 12 weeks of using the new HMEs compared with using the second-generation HMEs. An average decrease of 0.74 days (SD 0.70) in the number of days requiring chest physiotherapy was expected in patients using the new HMEs, compared with those using second-generation HMEs, after 12 weeks, and these patients were also expected to experience an average decrease of 34% (SD 32%) in pulmonary infections on a yearly basis. After 12 weeks of HME use, an average increase of 13% (SD 18%) in the number of social contacts and 33% (SD 30%) in overall QoL were expected in patients using the new HMEs compared with those using the second-generation HMEs. The use of the new HMEs was expected to result in 44% (SD 30%) of patients being more satisfied with their HME when compared with patients using the second-generation HMEs after 12 weeks. Experts expected an average of 53% (SD 28%) of patients breathing better after 12 weeks of using the new HMEs, and, on average, 48% (SD 25%) of patients were expected to experience a decrease in shortness of breath after 12 weeks of using the new HMEs. Experts believed that, on average, 59% (SD 19%) of patients will experience a decrease in tracheal dryness or irritation after 12 weeks of using the new HMEs. The average number of daily coughs after 12 weeks of using the new HMEs was expected to be 2.95 (SD 1.61) per patient, and the number of daily forced expectorations per patient after 12 weeks of new HME use was expected to be 2.46 (SD 1.42). Experts indicated that 48% (SD 22%) of patients will experience sleeping problems after 12 weeks of using the new HMEs. On average, 24% (SD 20%) of the patients were expected to experience psychosocial problems after 12 weeks of using the new HMEs.

**Table 2:** Average scores of expert judgments per effect variable.

Variables	Average scores (SD)	Experts (n=19), n (%)	SEE^a^ process
Tracheal climate	1.51 (0.24)	19 (100)	In total, 51% of patients will experience a decrease in tracheal dryness or irritation when using the new HMEs^b^ compared with second-generation HMEs after 12 weeks.
Mucus plugging	0.67 (0.32)	19 (100)	The percentage of decrease in patients experiencing mucus plug events when using the new HMEs compared with using the second-generation HMEs during the acute postoperative period (2 weeks) will be 33%.
Mucus production	0.47 (0.22)	19 (100)	After 12 weeks of using the new HMEs, 53% of patients will have decreased mucus production compared with no HME use.
Speech quality	1.25 (0.23)	18 (95)	Using the new HMEs for 12 weeks will result in 25% of patients having better speech quality compared with using the second-generation HMEs.
Physiotherapy	0.74 (0.70)	18 (95)	The average number of days requiring chest physiotherapy will decrease by 0.74 days after 12 weeks of using the new HMEs compared with using the second-generation HMEs.
Tracheobronchitis or pneumonia episodes	0.66 (0.32)	18 (95)	The percentage of decrease in pulmonary infections per year in patients using the new HMEs compared with those using the second-generation HMEs will be 34%.
Social contacts	1.13 (0.18)	18 (95)	The percentage of increase in the average number of social contacts in patients using the new HMEs compared with those using the second-generation HMEs after 12 weeks will be 13%.
QoL^c^	1.33 (0.30)	18 (95)	The percentage of increase in the overall QoL in patients using the new HMEs compared with those using the second-generation HMEs after 12 weeks will be 33%.
Patient satisfaction	1.44 (0.30)	18 (95)	Using the new HMEs will result in 44% of patients being more satisfied about their HME when compared with patients using the second-generation HMEs after 12 weeks.
Breathing	1.53 (0.28)	19 (100)	The use of the new HMEs will result in 53% of patients breathing better after 12 weeks of use.
Shortness of breath	1.48 (0.25)	19 (100)	After 12 weeks of using the new HMEs, 48% of patients will experience a decrease in shortness of breath.
Tracheal climate	1.59 (0.19)	19 (100)	After 12 weeks of using the new HMEs, 59% of patients will experience a decrease in tracheal dryness or irritation.
Coughing	2.95 (1.61)	19 (100)	The average amount of daily coughs per patient after 12 weeks of using the new HMEs will be 2.95.
Forced expectorations	2.46 (1.42)	19 (100)	The average number of daily forced expectorations per patient after 12 weeks of using the new HMEs will be 2.46.
Sleep quality	0.48 (0.22)	18 (95)	After 12 weeks of using the new HMEs, 48% of patients will experience sleeping problems.
Psychosocial aspects	0.24 (0.20)	18 (95)	After 12 weeks of using the new HMEs, 24% of patients will experience psychosocial problems.

^a^SEE: structured expert elicitation.

^b^HMEs: heat and moisture exchangers.

^c^QoL: quality of life.

## Discussion

### Principal Findings

The rapid review identified the benefits of the current HMEs regarding breathing effort, tracheal dryness and irritation, mucus production and plugging, inflammation and infection rates, sleep quality, QoL, and patient satisfaction, which were confirmed for the new HMEs by the experts during the interviews and SEE. There were some discrepancies regarding cough, forced expectorations, and psychosocial aspects: in the interviews, the experts mentioned that the new HMEs will improve these aspects, but this was not observed in SEE. In addition, the interviewed experts did not expect to see an effect of using the new HMEs on speech quality and the requirement of physiotherapy, but the SEE showed a positive effect for these variables. According to the interviewed experts, the new HME portfolio will presumably lead to higher adherence to HME use by patients and fewer health complaints and complications because of the improved HME properties, resulting in lower health care use (eg, reduced hospital readmissions).

### Comparison With Prior Work

The finding that HME use reduces coughing and forced expectorations in patients who underwent laryngectomy is comparable with the findings from previous studies [7,17], and the finding that patients who use an HME experience better sleep has also been reported before [13]. In line with our study, 2 studies reported improvements in coughing, the mean daily frequency of sputum production, forced expectoration, shortness of breath, sleep problems, and perceived voice quality [15,24]. In comparison with an earlier study [42], the SEE demonstrated that fewer patients (than reported) are expected to experience a decrease in tracheal dryness or irritation when using the new HMEs compared with when using the second-generation HMEs. However, when we directly asked the experts if they expected an increase or decrease in patients experiencing tracheal dryness or irritation with the new HMEs compared with the second-generation HMEs, they reported a decrease on average. In addition, compared with patients using the second-generation HMEs, the experts expected a reduction in patients experiencing mucus plug events in the postoperative period. In comparison, another study reported that approximately one-tenth of the patients using a second-generation HME developed mucus plugs during the postoperative period [43]. After 12 weeks of using the new HMEs, approximately half of the patients were expected to experience decreased mucus production compared with no HME use. This is more than what was reported for the first-generation HMEs [31] and less than what was reported for the second-generation HMEs [41]. Besides, on average, the breathing results expected with the use of the new HMEs were less improved than those reported with the use of the first-generation HMEs [40]. Experts expected that more patients will experience a decrease in shortness of breath with the use of the new HMEs compared with results obtained from the use of the first- and second-generation HMEs [31]. The experts also expected that the average number of daily coughs after 12 weeks of using the new HMEs will be slightly higher than that reported after using the second-generation HMEs [42]. The same applies to the average number of daily forced expectorations per patient [42]. The percentage of patients experiencing sleeping problems after 12 weeks of using the new HMEs is expected to be notably lower than that after using the first-generation HMEs [28]. The number of patients who are expected to experience psychosocial problems after using the new HMEs is similar to number of patients reported to experience psychosocial problems after using the first- and second-generation HMEs [31].

### Strengths and Limitations

To the best of our knowledge, this study is the first to investigate the expected effects of the new HMEs. A strength of this study is the use of a mixed methods approach, which helped to gain a deeper understanding of the effectiveness of HMEs than would be possible with the use of either approach alone. A second strength is the overall consensus among clinicians from leading German institutions on the benefits of the new HMEs compared with the current HMEs. Moreover, by using the literature and expert beliefs at an early stage in the life cycle of the new generation of HMEs, information can be generated to support decision-making [46].

A limitation to consider is the number of experts interviewed. Even though the sample size was small, consensus on the views on the new generation of HMEs was observed when interviewing the experts independently from each other. A second limitation is that most experts who completed the SEE survey did not yet have experience with prescribing the new HMEs. Hence, the current effect estimations are likely to be an underestimation of the true effect, as experts most likely filled in the survey conservatively. This point is addressed by our results; it is noticeable that the experts responded more conservatively when there was no comparison in the question than when a comparison was included. The categories tracheal dryness or irritation, mucus plug events, mucus production, speech quality, physiotherapy, pulmonary infections, social contacts, QoL, and patient satisfaction did contain a comparison, all showing results in favor of the new HMEs. The questions regarding the categories of breathing, shortness of breath, daily coughs, daily forced expectorations, sleeping problems, and psychosocial problems did not contain a comparator, and the results mostly showed a less favorable expected effect for the new HMEs, except for sleeping and psychosocial problems. Because the standard of care of all the experts was second-generation Provox HMEs, it might be possible that the experts subconsciously used these HMEs as comparators in their mind when answering the questions without comparators. In addition, some questions in the SEE survey contained categories in which experts could choose X or greater than X (ie, ≥X). When the experts entered weights in this category, we did not know exactly how much the expert meant by X or greater than X. We interpreted this by giving this category the value X plus 1 interval so that it would have a greater impact on the calculated mean. This could be an underestimation of the value given by the experts. As this category was barely used by the experts, it should not be regarded as a major limitation of the study.

### Future Considerations

It is recommended that the SEE survey be conducted once more after the experts have gained experience with the new HMEs, as the current effect estimations might be underestimated. To generate primary data on the actual use of the new HMEs by patients, it is recommended that a clinical study be set up to evaluate the effectiveness of the new HMEs. Both the perspectives of patients and health care providers need to be included in future studies on HMEs to gain a complete picture of the value of the new HMEs. Next, as in the current reimbursement landscape, a new medical device needs to be not only effective in improving patient outcomes but also cost-effective, and conducting an economic analysis is recommended.

### Conclusions

According to German experts, the use of the new HMEs with improved humidification levels by patients who underwent laryngectomy is expected to have additional (clinical) effectiveness on pulmonary health and QoL aspects compared with the currently available HMEs.

## Appendix

Multimedia Appendix 1 Topic guide for the interviews.

Multimedia Appendix 2 Average scores of expert judgements on effect variables.

Multimedia Appendix 3 Survey structured expert elicitation (SEE).

Multimedia Appendix 4 Average scores of expert judgements on effect variables.

## Abbreviations

EH: external humidifier
HME: heat and moisture exchanger
HRQoL: health-related quality of life
QoL: quality of life
RLR: rapid literature review
SEE: structured expert elicitation
TL: total laryngectomy

## References

[1] Balm AJ, van As-Brooks EJ (2007). Laryngeal and hypolaryngeal cancer: intervention approaches. Head and Neck Cancer: Treatment, Rehabilitation, and Outcomes.

[2] Wiskirska-Woźnica B, Leszczyńska M, Czerniejewska H, Jackowska J, Witold S, Swidziński (2011). Voice estimation in patients after reconstructive subtotal laryngectomy. Head Neck Oncol.

[3] Hilgers FJ, Ackerstaff AH, Aaronson NK, Schouwenburg PF, Van Zandwijk N (1990). Physical and psychosocial consequences of total laryngectomy. Clin Otolaryngol Allied Sci.

[4] Todisco T, Maurizi M, Paludetti G, Dottorini M, Merante F (1984). Laryngeal cancer: long-term follow-up of respiratory functions after laryngectomy. Respiration.

[5] Ackerstaff AH, Hilgers FJ, Aaronson NK, Balm AJ (1994). Communication, functional disorders and lifestyle changes after total laryngectomy. Clin Otolaryngol Allied Sci.

[6] Jones AS, Young PE, Hanafi ZB, Makura ZG, Fenton JE, Hughes JP (2003). A study of the effect of a resistive heat moisture exchanger (Trachinaze) on pulmonary function and blood gas tensions in patients who have undergone a laryngectomy: a randomized control trial of 50 patients studied over a 6-month period. Head Neck.

[7] Ackerstaff AH, Fuller D, Irvin M, Maccracken E, Gaziano J, Stachowiak L (2003). Multicenter study assessing effects of heat and moisture exchanger use on respiratory symptoms and voice quality in laryngectomized individuals. Otolaryngol Head Neck Surg.

[8] Boscolo-Rizzo P, Maronato F, Marchiori C, Gava A, Da Mosto MC (2008). Long-term quality of life after total laryngectomy and postoperative radiotherapy versus concurrent chemoradiotherapy for laryngeal preservation. Laryngoscope.

[9] Öztürk A, Mollaoğlu M (2013). Determination of problems in patients with post-laryngectomy. Scand J Psychol.

[10] Williams R, Rankin N, Smith T, Galler D, Seakins P (1996). Relationship between the humidity and temperature of inspired gas and the function of the airway mucosa. Crit Care Med.

[11] Hilgers FJ, Ackerstaff AH (2000). Comprehensive rehabilitation after total laryngectomy is more than voice alone. Folia Phoniatr Logop.

[12] van den Boer C, van Harten MC, Hilgers FJ, van den Brekel MW, Retèl VP (2014). Incidence of severe tracheobronchitis and pneumonia in laryngectomized patients: a retrospective clinical study and a European-wide survey among head and neck surgeons. Eur Arch Otorhinolaryngol.

[13] Retèl VP, van den Boer C, Steuten LM, Okła S, Hilgers FJ, van den Brekel MW (2015). Cost-effectiveness of heat and moisture exchangers compared to usual care for pulmonary rehabilitation after total laryngectomy in Poland. Eur Arch Otorhinolaryngol.

[14] Zuur JK, Muller SH, Vincent A, Sinaasappel M, de Jongh FH, Hilgers FJ (2009). The influence of a heat and moisture exchanger on tracheal climate in a cold environment. Med Eng Phys.

[15] Ackerstaff AH, Hilgers FJ, Aaronson NK, Balm AJ, van Zandwijk N (1993). Improvements in respiratory and psychosocial functioning following total laryngectomy by the use of a heat and moisture exchanger. Ann Otol Rhinol Laryngol.

[16] Brook I, Bogaardt H, van As-Brooks C (2013). Long-term use of heat and moisture exchangers among laryngectomees: medical, social, and psychological patterns. Ann Otol Rhinol Laryngol.

[17] Hilgers FJ, Aaronson NK, Ackerstaff AH, Schouwenburg PF, van Zandwikj N (1991). The influence of a heat and moisture exchanger (HME) on the respiratory symptoms after total laryngectomy. Clin Otolaryngol Allied Sci.

[18] Ackerstaff AH, Hilgers FJ, Aaronson NK, Schouwenburg PF, van Zandwijk N (1990). [Physical and psychosocial sequelae of total larynx extirpation and the use of a heat and moisture exchanger]. Ned Tijdschr Geneeskd.

[19] Zuur JK, Muller SH, Vincent A, Sinaasappel M, de Jongh FH, Hilgers FJ (2008). Assessment of tracheal temperature and humidity in laryngectomized individuals and the influence of a heat and moisture exchanger on tracheal climate. Head Neck.

[20] Scheenstra RJ, Muller SH, Hilgers FJ (2011). Endotracheal temperature and humidity in laryngectomized patients in a warm and dry environment and the effect of a heat and moisture exchanger. Head Neck.

[21] Keck T, Dürr J, Leiacker R, Rettinger G, Rozsasi A (2005). Tracheal climate in laryngectomees after use of a heat and moisture exchanger. Laryngoscope.

[22] Rozsasi A, Leiacker R, Fischer Y, Keck T (2006). Influence of passive humidification on respiratory heat loss in tracheotomized patients. Head Neck.

[23] Scheenstra RJ, Muller SH, Vincent A, Ackerstaff AH, Jacobi I, Hilgers FJ (2010). Short-term endotracheal climate changes and clinical effects of a heat and moisture exchanger with an integrated electrostatic virus and bacterial filter developed for laryngectomized individuals. Acta Otolaryngol.

[24] Ackerstaff AH, Hilgers FJ, Aaronson NK, De Boer MF, Meeuwis CA, Knegt PP, Spoelstra HA, Van Zandwijk N, Balm AJ (1995). Heat and moisture exchangers as a treatment option in the post-operative rehabilitation of laryngectomized patients. Clin Otolaryngol Allied Sci.

[25] Hilgers FJ, Ackerstaff AH, Balm AJ, Gregor RT (1996). A new heat and moisture exchanger with speech valve (Provox stomafilter). Clin Otolaryngol Allied Sci.

[26] Ackerstaff AH, Hilgers FJ, Balm AJ, Tan IB (1998). Long-term compliance of laryngectomized patients with a specialized pulmonary rehabilitation device: Provox Stomafilter. Laryngoscope.

[27] Herranz González-Botas J, Suárez T, García Carreira B, Martínez Morán A (2001). [Experience with the HME-Provox Stomafilter in laryngectomized patients]. Acta Otorrinolaringol Esp.

[28] Bień S, Okła S, van As-Brooks CJ, Ackerstaff AH (2010). The effect of a Heat and Moisture Exchanger (Provox HME) on pulmonary protection after total laryngectomy: a randomized controlled study. Eur Arch Otorhinolaryngol.

[29] Mérol JC, Charpiot A, Langagne T, Hémar P, Ackerstaff AH, Hilgers FJ (2012). Randomized controlled trial on postoperative pulmonary humidification after total laryngectomy: external humidifier versus heat and moisture exchanger. Laryngoscope.

[30] van den Boer C, Muller SH, Vincent AD, van den Brekel MW, Hilgers FJ (2014). Ex vivo assessment and validation of water exchange performance of 23 heat and moisture exchangers for laryngectomized patients. Respir Care.

[31] Herranz J, Espiño MA, Morado CO (2013). Pulmonary rehabilitation after total laryngectomy: a randomized cross-over clinical trial comparing two different heat and moisture exchangers (HMEs). Eur Arch Otorhinolaryngol.

[32] Leemans M, van Sluis KE, van Son RJ, van den Brekel MW (2020). Interaction of functional and participation issues on quality of life after total laryngectomy. Laryngoscope Investig Otolaryngol.

[33] Pedemonte-Sarrias G, Villatoro-Sologaistoa JC, Ale-Inostroza P, López-Vilas M, León-Vintró X, Quer-Agustí M (2013). Chronic adherence to heat and moisture exchanger use in laryngectomized patients. Acta Otorrinolaringol Esp.

[34] Soares MO, Sharples L, Morton A, Claxton K, Bojke L (2018). Experiences of structured elicitation for model-based cost-effectiveness analyses. Value Health.

[35] King VJ, Stevens A, Nussbaumer-Streit B, Kamel C, Garritty C (2022). Paper 2: performing rapid reviews. Syst Rev.

[36] Leenaars C, Tsaioun K, Stafleu F, Rooney K, Meijboom F, Ritskes-Hoitinga M, Bleich A (2021). Reviewing the animal literature: how to describe and choose between different types of literature reviews. Lab Anim.

[37] Johnson SR, Tomlinson GA, Hawker GA, Granton JT, Grosbein HA, Feldman BM (2010). A valid and reliable belief elicitation method for Bayesian priors. J Clin Epidemiol.

[38] Vizzotto ADB, de Oliveira AM, Elkis H, Cordeiro Q, Buchain PC, Gellman MD, Turner JR (2013). Psychosocial Characteristics. Springer.

[39] Tracheobronchitis. Healthline.

[40] Dassonville O, Mérol JC, Bozec A, Swierkosz F, Santini J, Chaïs A, Marcy PY, Giacchero P, Chamorey E, Poissonnet G (2011). Randomised, multi-centre study of the usefulness of the heat and moisture exchanger (Provox HME®) in laryngectomised patients. Eur Arch Otorhinolaryngol.

[41] Macri GF, Bogaardt H, Parrilla C, Minni A, D'Alatri L, de Vincentiis M, Greco A, Paludetti G (2016). Patients' experiences with HMEs and attachments after total laryngectomy. Clin Otolaryngol.

[42] Parrilla C, Minni A, Bogaardt H, Macri GF, Battista M, Roukos R, Pandolfini M, Ruoppolo G, Paludetti G, D'Alatri L, de Vincentiis M (2015). Pulmonary rehabilitation after total laryngectomy: a multicenter time-series clinical trial evaluating the Provox XtraHME in HME-naïve patients. Ann Otol Rhinol Laryngol.

[43] Ebersole B, Moran K, Gou J, Ridge J, Schiech L, Liu JC, Lango M (2020). Heat and moisture exchanger cassettes: results of a quality/safety initiative to reduce postoperative mucus plugging after total laryngectomy. Head Neck.

[44] Foreman A, De Santis RJ, Sultanov F, Enepekides DJ, Higgins KM (2016). Heat and moisture exchanger use reduces in-hospital complications following total laryngectomy: a case-control study. J Otolaryngol Head Neck Surg.

[45] Act on medical-scientific research with humans. Central Committee on Research Involving Human Subjects.

[46] Ijzerman MJ, Steuten LM (2011). Early assessment of medical technologies to inform product development and market access: a review of methods and applications. Appl Health Econ Health Policy.

