# Supplementary material for: New Heat and Moisture Exchangers for Laryngectomized Patients in Germany: Mixed Methods Study on the Expected Effectiveness
Source: JMIR Form Res. 2023 Jan 11;7:e36401. doi: 10.2196/36401 (PMC9878367; doi:10.2196/36401)
Supplement: Multimedia Appendix 1 [file formative_v7i1e36401_app1.pdf]

Multimedia Appendix 1 – Topic guide for the interviews

1. Introduction round
2. Introduction new HMEs (Provox Life)
3. Main questions

Compliance

- What factors play a role in the compliance of patients?
- How do better products influence compliance? Is it expected that a better product will lead to higher compliance?
- To what extent does higher compliance lead to fewer complications?
- How often are problems or dissatisfaction with HME use the cause for non-compliance?

Breathing resistance

- RLR: Breathing was equal or less strenuous in HME group when compared to control group
- What are the consequences of patients having difficulties with breathing resistance?
- To what kind of care and/or complications does this lead to?
- To what extent might this be changed by the use of Provox Life?

Cough and forced expectorations

- RLR: Decrease in coughing episodes, daily coughs, and in daily forced expectorations in HME group (when compared to control)
- To what kind of complications can coughing lead to? What type of care is needed consequently?
- How does this affect quality of life?
- Do you expect that cough episodes/ forced expectorations might be changed by the use of Provox Life?

Tracheal dryness/irritation

- RLR: Improvement in tracheal climate and less tracheal dryness/irritation
- What are the consequences of patients having tracheal dryness/irritation?
- To what kind of care and/or complications does this lead to?
- Does the effect on tracheal climate depends on which HME the patient uses?

- To what extent will this be changed by the use of Provox Life?

Mucus production/plugs

- RLR: 1) Less mucus production/plugging in HME group compared to control group. 2)Rate of mucus plugging lower in HME group
- What are the consequences of patients having mucus plugs?
- How does this affect quality of life?
- To what kind of care and/or complications does this lead to?
- To what extent will this be changed by the use of Provox Life?

Speech intelligibility and voice quality

- RLR: Improved voice quality by HME use (compared to control)
- How important is speech intelligibility and voice quality found by the patients when using a HME?
- What kind of care is related to patients having difficulties with speech?
- To what extent will this be changed by the use of Provox Life?

Sleep quality

- RLR: Fewer sleep disturbances/improved sleep in HME group
- What are the consequences of patients having difficulties with sleeping?
- To what kind of care and/or complications does this lead to?
- What part of the patients uses sleep medication?
- To what extent might sleep difficulties be influenced by the use of Provox Life?

Physiotherapy

- RLR: Decrease in number of days (chest) physiotherapy is required in HME group
- What percentage of the patients needs physiotherapy after laryngectomy?
- What type of patients are these?
- To what extent might the need for physiotherapy be changed by the use of Provox Life?

Quality of life/satisfaction

- RLR: 1) Increase in quality of life in HME group. 2) Patient satisfaction is increased/high with HME use. 3) When using a hands-free device, patients tend to have more frequent social contacts

- What other factors related to HME use are relevant for the quality of life for patients? Are there any other effects/complications that play a role and have not yet been mentioned?

Psychological health

- What percentage of the laryngectomized patients has psychological issues?
- To what kind of care and/or complications does this lead to?
- To what extent will this be changed by the use of Provox Life?

Participation in society

- What part of the laryngectomized patients is employed?
- To what extent does HME use has an impact on the number of sick days or influence the possibility to go back to work?

To what extent might the use of Provox Life have an effect on:

- Tracheobronchitis and pneunomia episodes;
- (length of) hospital stay;
- Hospital readmission.
