# Supplementary material for: New Heat and Moisture Exchangers for Laryngectomized Patients in Germany: Mixed Methods Study on the Expected Effectiveness
Source: JMIR Form Res. 2023 Jan 11;7:e36401. doi: 10.2196/36401 (PMC9878367; doi:10.2196/36401)
Supplement: Multimedia Appendix 2 [file formative_v7i1e36401_app2.pdf]

Multimedia Appendix 2 – Calculation example (fictional answers)

Below an example of a calculation is provided on a question indicating either an increase or decrease of an effect variable (fictional data).

Question: please enter weights in the two tables below which indicate what percentage of patients you expect to either breathe worse (Table 1) or better (Table 2) after 12 weeks of Provox Life use.

First, weights were entered by the experts (Table 1 and 2)

Table 1. Use of Provox Life results in ..% of patients breathing worse

% patients	100-91	90-80	80-71	70-61	60-51	50-40	40-31	30-21	20-11	10-0
Weight	0	0	0	0	0	5	5	5	5	10

Table 2. Use of Provox Life results in ..% of patients breathing better

% patients	0-10	11-20	21-30	31-40	41-50	51-60	61-70	71-80	81-90	91-100	TOTAL
Weight	10	10	30	20	0	0	0	0	0	0	100

Then each interval was given a score (Table 3 and 4).

Table 3. 1 - average percentage to indicate the decline (breathing worse)

Interval	100-91	90-80	80-71	70-61	60-51	50-40	40-31	30-21	20-11	10-0
Score	0.05	0.15	0.25	0.35	0.45	0.55	0.65	0.75	0.85	0.95

Table 4. 1 + average percentage to indicate the improvement (breathing better)

Interval	0-10	11-20	21-30	31-40	41-50	51-60	61-70	71-80	81-90	91-100
Score	1.05	1.15	1.25	1.35	1.45	1.55	1.65	1.75	1.85	1.95

Then the score was multiplied by the given weight (Table 5 and 6)

Table 5. Use of Provox Life results in ..% of patients breathing worse:

Score	0.05	0.15	0.25	0.35	0.45	0.55	0.65	0.75	0.85	0.95
Weight	0	0	0	0	0	5	5	5	5	10
Score x Weight =	0	0	0	0	0	2.75	3.25	3.75	4.25	9.50

Table 6. Use of Provox Life results in ..% of patients breathing better

Score	1.05	1.15	1.25	1.35	1.45	1.55	1.65	1.75	1.85	1.95	TOTAL
Weight	10	10	30	20	0	0	0	0	0	0	100
Score x Weight =	10.50	11.50	37.5	27	0	0	0	0	0	0	110

Final indicator = total (score x weight) / total weights = 110 / 100 = 1.10

Result: This expert beliefs that the use of Provox Life results in 10% of patients breathing better.

(Remember: an outcome of 0-1 indicates the expected decline, and 1-2 the expected improvement)
