# Supplementary material for: New Heat and Moisture Exchangers for Laryngectomized Patients in Germany: Mixed Methods Study on the Expected Effectiveness
Source: JMIR Form Res. 2023 Jan 11;7:e36401. doi: 10.2196/36401 (PMC9878367; doi:10.2196/36401)
Supplement: Multimedia Appendix 3 [file formative_v7i1e36401_app3.pdf]

Multimedia Appendix 3 - Survey SEE

Introduction

Dear [name expert],

On behalf of Atos Medical, we kindly invite you to fill out this survey.

The objective of this study is to elicit your judgement about the effectiveness of Provox Life and its impact on healthcare use. In doing so, you will be asked to express your judgements in a statistical, quantitative form.

Since this survey is intended for experts who are familiar with Provox Life and who have been invited to participate, background information about Provox Life was shared with you.
Before answering the questions in this survey, please make sure to read the whitepaper and watch the video about Provox Life. If you have **not received** a whitepaper and a short video from Atos Medical, please contact [name researcher] via [email] to receive this information before you fill out this survey.

This survey consists of two sections with sets of questions. Every set of questions is introduced by a short text. Before you start answering, please read the introduction text carefully as well as the question and answering categories. On average, it will take about 30-40 minutes to fill out this survey. Please fill out this survey from start till end.

Be aware that all answers will be processed anonymously; during analysis and reporting no answers will be related to any individual. If you have any concerns and/or questions, please contact [name] via email: [address]

Thank you very much for your time and answers!

Please enter your contact details in the boxes below. As mentioned before; all of your responses will be processed anonymously, during analysis and reporting no answers will be related to any individual.

PART I

Background characteristics

This set of questions aims to provide us a picture of your professional expertise in the field of laryngeal cancer and your experience with Heat and Moisture Exchangers (HMEs).

1. What is your current profession?
   - o Head and neck surgeon
   - o Otolaryngologists/ENT physician
   - o Physiotherapist
   - o Speech Language Pathologist
   - o Other, namely: ________________________________

2. How many years of experience do you have with providing care to patients with laryngeal cancer?

   ______ years

3. How many years of experience do you have with providing care to total laryngectomy patients?

   ______ years

4. How many years of experience do you have with prescribing and/or monitoring the use of HMEs?

   ______ years

5. How many years of experience do you have as a scientific researcher in the field of laryngeal cancer?

   ______ years

6. Did your read the white paper on Provox Life and watch the video on Provox Life?
   - o Yes
   - o No (you have reached the end of this survey)

7. What is your experience with Provox Life?
    - I have heard from Provox Life but have not yet prescribed it.
    - I have prescribed Provox Life at least once.

PART II

Based on scientific literature we quantified the effects of HMEs on **14 different outcomes** which are of interest after a total laryngectomy. We are interested in your estimation of the quantified effects for Provox Life.

Before asking you to provide your judgement, we first show you an example of how to answer the questions in this section. Please read the example carefully to fully understand the nature of this type of questions.

<u>EXAMPLE:</u>

Imagine a semi-final match at EURO 2020, the UEFA European Championship, with Italy playing against Spain. Until now, the national teams of these two countries have played 37 matches against each other. Their last game (in 2017) was won by Spain with 3 goals for Spain against 0 for Italy.

A sports journalist from Sweden was asked to give weights to the number of goals in favour of either Italy or Spain assuming these countries would play the semi-final at EURO 2020.

He was instructed to enter his weights of belief in each of the possible number of goals in favour of either Italy or Spain as shown in the table below. The stronger he believes that the number of goals in favour of either Spain or Italy will truly lie in a given cell, the greater should be his weight for that number. If he believes it is impossible that the actual number of goals lies in a given cell, his weight should be zero. His weights should add up to 100.

Based on the provided information, the Swedish journalist has answered the question (answers in blue). He believes most strongly that Italy and Spain will play equal (20+20=40). Also, he thinks either Italy or Spain may win with 1 up to 4 goals in favour of one of them. The weights given add up to 100.

Table 1:

Number of goals **in favour of Italy**

Goals	6	5	4	3	2	1	0
Weight	0	0	5	5	10	10	20

Table 2:

Number of goals **in favour of Spain**

Goals	0	1	2	3	4	5	6	TOTAL
Weight	20	10	10	5	5	0	0	100

1. **Breathing**

After a total laryngectomy patients experience difficulties with breathing. A study reported that 71% of patients responded positively to the question "Are you breathing better?" after 6 weeks of HME use, and at 3 months 88%. However, no statistically significant difference was reported in this study [40].

1A. Please enter weights in the two tables below which indicate what percentage of patients you expect to either breathe worse (table 1) or better (table 2) after 12 weeks of Provox Life use.

Enter your weights of belief in each of the possible intervals of percentage shown in the table below. The stronger you believe that the percentage of patients with more or less difficult in breathing will truly lie in a given interval, the greater should be your weight for that interval (maximum weight of 100). If you believe the percentage of patients with more or less difficulty in breathing does not lie in a given interval, your weight should be zero for that specific interval. Please insert an answer in every row.

1B. Please sum your weights across both tables in the final row of the second table below. The total sum should be 100.

Please fill in your answers in the tables below (use your mouse to go to the next box):

Table 1:

Use of Provox Life results in ..% of patients **breathing worse**:

% Patients	100-91	90-80	80-71	70-61	60-51	50-40	40-31	30-21	20-11	10-0
Weight										

Table 2:
Use of Provox Life results in .. % of patients **breathing better**:

% Patients	0-10	11-20	21-30	31-40	41-50	51-60	61-70	71-80	81-90	91-100	**TOTAL** (should add up to 100)
Weight											

2. Shortness of breath

The results regarding shortness of breath when using a HME are:

- a statistically significant decrease in shortness of breath was demonstrated with a baseline value of 5.7 and a value of 3.8 after 6 and 12 weeks of Provox XtraHME (second generation) use (p<0.0001) with the Structured Questionnaires [42];
- after 12 weeks of Provox XtraHME (second generation) use, 3.4% of patients found it more difficult to breathe through the HME, 24.1% of patients felt no difference and 72.4% of patients found breathing through the HME less difficult (p=0.002) [41].
- non-HME users, Provox Micron HMEs (first generation) users, and Provox HME (first generation) users scored a 4.6, 4.9, and 4.3 respectively on the Shortness of Breath category from the Quality-of-Life Questionnaire (p=0.363) [16].

2A. Please enter weights in the two tables below which indicate what percentage of patients you expect to experience either an increase (table 1) or a decrease (table 2) in shortness of breath after 12 weeks when they use Provox Life.

Enter your weights of belief in each of the possible intervals of percentage shown in the table below. The stronger you believe that the percentage of patients with an increase or decrease in shortness of breath will truly lie in a given interval, the greater should be your weight for that interval (maximum weight of 100). If you believe the percentage of patients with an increase or decrease in shortness of breath does not lie in a given interval, your weight should be zero for that specific interval. Please insert an answer in every row.

2B. Please sum your weights across both tables in the final row of the second table below. The total sum should be 100.

Please fill in your answers in the tables below (use your mouse to go to the next box):

Table 1:
Percentage of patients experiencing an i**ncrease in shortness of breath** when using Provox Life:

% Patients	100-91	90-80	80-71	70-61	60-51	50-40	40-31	30-21	20-11	10-0
Weight										

Table 2:
Percentage of patients experiencing a **decrease in shortness of breath** when using Provox Life:

% Patients	0-10	11-20	21-30	31-40	41-50	51-60	61-70	71-80	81-90	91-100	**TOTAL** (should add up to 100)

3. **Tracheal climate**

An HME helps compensate for the humidification deficit in total laryngectomy patients. Inside the HMEs is a foam disc impregnated with hygroscopic salt, which captures heat and moisture from exhaled air to warm and moisturize the inhaled air[1].

After 2 weeks, 60.0% of the patients (using Provox XtraHME (second generation)) reported less tracheal dryness/irritation, and 40.0% reported no changes, compared to no HME use at baseline. After 12 weeks, 82.8% reported less irritation, 13.8% reported no changes, 3.4% reported more irritation (p=0.013) [42].

3A. Please enter weights in the two tables below which indicate what percentage of patients you expect to experience either an increase (table 1) or a decrease (table 2) in tracheal dryness/irritation after 12 weeks when they use Provox Life.

Enter your weights of belief in each of the possible intervals of percentage shown in the table below. The stronger you believe that the percentage of patients with an increase or decrease in tracheal dryness/irritation will truly lie in a given interval, the greater should be your weight for that interval

[1] Atos Medical, unpublished data, 2021

(maximum weight of 100). If you believe the percentage of patients with an increase or decrease in tracheal dryness/irritation does not lie in a given interval, your weight should be zero for that specific interval. Please insert an answer in every row.

3B. Please sum your weights across both tables in the final row of the second table below. The total sum should be 100.

Please fill in your answers in the tables below (use your mouse to go to the next box)**:**

Table 1:
Percentage of patients experiencing an **increase in tracheal dryness/irritation** when using Provox Life:

% Patients	100-91	90-80	80-71	70-61	60-51	50-40	40-31	30-21	20-11	10-0
Weight										

Table 2:
Percentage of patients experiencing a **decrease in tracheal dryness/irritation** when using Provox Life:

% Patients	0-10	11-20	21-30	31-40	41-50	51-60	61-70	71-80	81-90	91-100	**TOTAL** (should add up to 100)
Weight											

A study that compared first (Provox Normal and HiFlow) and second generations HMEs (Provox XtraMoist and XtraFlow) found that patients reported statistically significant less tracheal dryness with the second generation HMEs (38% less tracheal dryness) than with the first generation HMEs (14% less tracheal dryness) ($p = 0.039$) after 12 weeks of use [31].

3C. Please enter weights in the two tables below which indicate what percentage of patients you expect to experience an increase (table 1) or decrease (table 2) in tracheal dryness and irritation when comparing Provox Life to second generation HMEs (XtraMoist and XtraFlow) after 12 weeks of use.

Enter your weights of belief in each of the possible intervals of percentage shown in the table below. The stronger you believe that the percentage of patients with an increase or decrease in tracheal dryness/irritation will truly lie in a given interval, the greater should be your weight for that interval (maximum weight of 100). If you believe the percentage of patients with an increase or decrease in tracheal dryness/irritation does not lie in a given interval, your weight should be zero for that specific interval. Please insert an answer in every row.

3D. Please sum your weights across both tables in the final row of the second table below. The total sum should be 100.

Please fill in your answers in the tables below (use your mouse to go to the next box):

Table 1:

Percentage of patients experiencing an **increase in tracheal dryness/irritation** when using Provox Life compared to second generation HMEs (XtraMoist/XtraFlow):

% Patients	100-91	90-80	80-71	70-61	60-51	50-40	40-31	30-21	20-11	10-0
Weight										

Table 2:

Percentage of patients experiencing a **decrease in tracheal dryness/irritation** when using Provox Life compared to second generation HMEs (XtraMoist/XtraFlow):

% Patients	0-10	11-20	21-30	31-40	41-50	51-60	61-70	71-80	81-90	91-100	TOTAL (should add up to 100)
Weight											

4. Mucus production/plugging

Direct inhalation of dry and cold air into the lower airways can both stimulate the production of mucus and reduce the capacity of mucociliary clearance, which is important for clearing the airways from mucus and inhaled particles, bacteria, and viruses[2]. Use of an HME should decrease mucus production and mucus plugging.

After 2 weeks of Provox XtraHME (second generation) use, there was statistically significant less mucus production in patients [42]. A study comparing external humidifier (EH) use with Provox XtraHME (second generation) found that 50% of the patients using EH developed mucus plugs during the acute postoperative period, compared to 11% of the patients using the HME (p=0.01) [43].

[2]Atos Medical, unpublished data, 2021

4A. Please enter weights in the two tables below which indicate what percentage of patients you expect to experience either a decrease (table 1) or increase (table 2) in mucus plug events during the acute postoperative period (2 weeks) for Provox Life use compared to Provox XtraHME (second generation) use.

Enter your weights of belief in each of the possible intervals of percentage shown in the table below. The stronger you believe that the percentage of patients with an increase or decrease in mucus plus events will truly lie in a given interval, the greater should be your weight for that interval (maximum weight of 100). If you believe the percentage of patients with an increase or decrease in mucus plug events does not lie in a given interval, your weight should be zero for that specific interval. Please insert an answer in every row.

4B. Please sum your weights across both tables in the final row of the second table below. The total sum should be 100.

Please fill in your answers in the tables below (use your mouse to go to the next box):

Table 1:

Percentage of **decrease in patients experiencing mucus plug events** when using Provox Life compared to patients using Provox XtraHME (second generation) after the acute postoperative period (2 weeks):

% Patients	100-91	90-80	80-71	70-61	60-51	50-40	40-31	30-21	20-11	10-0
Weight										

Table 2:

Percentage of **increase in patients experiencing mucus plug events** when using Provox Life compared to patients Provox XtraHME (second generation) after the acute postoperative period (2 weeks):

% Patients	0-10	11-20	21-30	31-40	41-50	51-60	61-70	71-80	81-90	91-100	**TOTAL** (should add up to 100)
Weight											

After 12 weeks of use, 36% of the patients reported less mucus production with the first generation HME (Provox HME), 26% with the second generation HME (Provox XtraHME) and 41% reported no difference (p = 0.162) [31].
After 12 weeks of Provox Xtra HME (second generation) use, 79.3% of patient reported less mucus production, 6.9% more mucus production and 13.8% the same mucus production (p = 0.368) as compared to no HME use [41].

4C. Please enter weights in the two tables below which indicate what percentage of patients you expect to experience decreased (table 1) or increased (table 2) mucus production compared to no HME use after 12 weeks of Provox Life use?

Enter your weights of belief in each of the possible intervals of percentage shown in the table below. The stronger you believe that the percentage of patients experiencing more or less mucus production will truly lie in a given interval, the greater should be your weight for that interval (maximum weight of 100). If you believe the percentage of patients with an increase or decrease in mucus production does not lie in a given interval, your weight should be zero for that specific interval. Please insert an answer in every row.

4D. Please sum your weights across both tables in the final row of the second table below. The total sum should be 100.

Please fill in your answers in the tables below (use your mouse to go to the next box)**:**

Table 1:

Percentage of patients with **decreased mucus production** using Provox Life compared to no HME use after 12 weeks:

% Patients	100-91	90-80	80-71	70-61	60-51	50-40	40-31	30-21	20-11	10-0
Weight										

Table 2:

Percentage of patients with **increased mucus production** using Provox Life compared to no HME use after 12 weeks:

% Patients	0-10	11-20	21-30	31-40	41-50	51-60	61-70	71-80	81-90	91-100	**TOTAL** (should add up to 100)
Weight											

5. **Coughing**

One of the reported effects after total laryngectomy is frequent (involuntary) daily coughing, due to insufficient humidification[3]. We are interested in the effect of HME use on frequent daily coughing.
A study [42] which looked at clinical outcomes after Provox XtraHME (second generation) use reported that at baseline (control), after 2, 6, and 12 weeks of Provox XtraHME (second generation) use the average number of daily coughs was 8.8, 4.6, 3.5 and 2.4 respectively ($p<0.001$).

5A. Please enter weights in the table below which indicate how many daily coughs you expect patients to experience on average after 12 weeks Provox Life use?

Enter your weights of belief in each of the possible intervals shown in the table below. The stronger you believe that the average amount of daily coughs per patients will truly lie in a given interval, the greater should be your weight for that interval (maximum weight of 100). If you believe the average amount of daily coughs per patient does not lie in a given interval, your weight should be zero for that specific interval. Please insert an answer in every row.

5B. Please add your weights in the final column in the table below. These weights should add up to 100.

Please fill in your answers in this table (use your mouse to go to the next box)**:**

[3] Atos Medical, unpublished data, 2021

Average amount of **daily coughs** per patients after 12 weeks of Provox Life use:

Daily coughs	0-1.0	1.1-2.0	2.1-3.0	3.1-4.0	4.1-5.0	5.1-6.0	6.1-7.0	7.1-8.0	8.1-9.0	9.1-10.0	≥10.1	TOTAL (should add up to 100)
Weight												

6. Forced expectorations

Direct inhalation of dry and cold air into the lower airways can both stimulate the production of mucus and reduce the capacity of mucociliary clearance, which is important for clearing the airways from mucus and inhaled particles, bacteria, and viruses. Consequently, total laryngectomy patients often suffer from frequent forced expectorations to clear the airways[4].

A statistically significant decrease in the number of daily forced expectorations in Provox XtraHME (second generation) group versus control (p<.0001) was reported. Moreover, at baseline (control), after 2, 6, and 12 weeks of Provox XtraHME (seconed generation) use the average number of daily forced expectorations was 6.3, 3.0, 2.3 and 1.9 respectively [42].
The frequency of mucus expectoration for clearing the trachea was statistically significant lower in the Provox HME (first generation) arm (p<0.001). In the external humidifier (EH) group, the mean frequency of mucus expectoration was 5.5 times per day and in the Provox HME (first generation) group was 2.5 times per day [29].

6A. Please enter weights in the table below which indicate how many daily forced expectorations you expect patients to experience on average after 12 weeks Provox Life use?

Enter your weights of belief in each of the possible intervals shown in the table below. The stronger you believe that the average amount of daily forced expectorations per patients will truly lie in a given interval, the greater should be your weight for that interval (maximum weight of 100). If you believe the average amount of daily forced expectorations of patients lies in a given interval your weight should be zero for that specific interval. Please insert an answer in every row.

[4] Atos Medical, unpublished data, 2021

6B. **Please add your weights in the final column in the table below**. These weights should add up to 100.

Please fill in your answers in this table (use your mouse to go to the next box):

Average number of daily **forced expectorations** per patients after 12 weeks of Provox Life use:

Average number of daily forced expectorations	0-1.0	1.1-2.0	2.1-3.0	3.1-4.0	4.1-5.0	5.1-6.0	6.1-7.0	7.1-8.0	8.1-9.0	9.1-10.0	≥10.1	TOTAL (should add up to 100)
Weight												

7. **Sleep quality**

An example of a sleep problem among laryngectomy patients is waking up from coughing at night[5]. Multiple studies looked at the impact on sleep quality due to HME use:

- a statistically significant improvement was reported in sleep quality with a baseline value of 7.1 and a value of 6.2 (a lower score indicates less burden for the patient) after 12 weeks of Provox XtraHME (second generation) use (p=0.004) on the Structured Questionnaires [42].
- in the control group of another study, almost all patients (97.5%) had sleeping problems and this did not change over time. In the full compliance Provox HME (first generation) group, 79% of the patients had sleeping problems at baseline and 72% had this problem after 3 months. This reduction was not statistically significant [28].

7A. **Please enter weights in the table below** which indicate what percentage of patients you expect to experience sleeping problems after 12 weeks of Provox Life use?

Enter your weights of belief in each of the possible intervals shown in the table below. The stronger you believe that the percentage of patients experiencing sleeping problems will truly lie in a given interval, the greater should be your weight for that interval (maximum weight of 100). If you believe the percentage of patients with sleeping problems does not lie in a given interval, your weight should be zero for that specific interval. Please insert an answer in every row.

[5] Atos Medical, unpublished data, 2021

7B. Please add your weights in the final column of the table below. These weights should add up to 100.

Please fill in your answers in this table (use your mouse to go to the next box):

Percentage of patients experiencing **sleeping problems** after 12 weeks of Provox Life use.

% Patients	0-10	11-20	21-30	31-40	41-50	51-60	61-70	71-80	81-90	91-100	**TOTAL** (should add up to 100)
Weight											

8. Speech quality

A secondary function of HMEs is providing means for stoma occlusion to enable tracheoesophageal speech via a voice prosthesis, which for some HME models can be accomplished in a hands-free manner[6].

However, results from literature differ on the effect of HME use on speech quality:

- a statistically significant improvement in speech quality with a baseline value (HME naïve) of 12.3 versus 10.3 after 12 weeks of Provox XtraHME (second generation) use ($p<0.0001$) (a lower score indicates less burden for the patient) on the Structured Questionnaires [42].
- After 6 weeks of use, no difference was reported in speech intelligibility and voice ($p=0.739$) between Provox HME (first generation) and Provox XtraHME (second generation) [31].

8A. Please enter weights in the two tables below which indicate what percentage of patients you expect to experience either a worse (table 1) or better (table 2) speech quality after 12 weeks of Provox Life use compared to Provox XtraHME (second generation) use.

Enter your weights of belief in each of the possible intervals of percentage shown in the table below. The stronger you believe that the percentage of patients with a better or worse speech quality will truly lie in a given interval, the greater should be your weight for that interval (maximum weight of 100). If you believe the percentage of patients with a better or worse speech quality does not lie in a given

[6] Atos Medical, unpublished data, 2021

interval, your weight should be zero for that specific interval. Please insert an answer in every row.

8B. Please sum your weights across both tables in the final row of the second table below. The total sum should be 100.

Please fill in your answers in this table (use your mouse to go to the next box):

Table 1:

12 weeks use of Provox Life results in ..% of patients with **worse speech quality** compared to Provox XtraHME (second generation) use:

% Patients	100-91	90-80	80-71	70-61	60-51	50-40	40-31	30-21	20-11	10-0
Weight										

Table 2:

12 weeks use of Provox Life results in ..% of patients with **better speech quality** compared to Provox XtraHME (second generation) use:

% Patients	0-10	11-20	21-30	31-40	41-50	51-60	61-70	71-80	81-90	91-100	**TOTAL** (should add up to 100)
Weight											

9. Psychosocial aspects

Psychosocial characteristics is a term used to describe the influences of social factors on an individual's mental health and behaviour [38]. Many total laryngectomy patients suffer from both physical and psychosocial problems, which can have a major impact on their quality of life (QoL). Patients reported no differences concerning psychosocial aspects in one study. Both for the first and second generation HMEs over 75% of the patients reported to have no problems, socially or psychologically [31].

In another study, most patients (80–90%) at baseline reported no or only slight problems with anxiety and depression, which had statistically significantly increased in the control group (p=0.003) (no HME

use), whereas in the Provox HME (first generation) groups no statistically significant changes were found [28].
At last, a study found a statistically significant reduction in psychological stress with a baseline value of 7.1 versus 5.1 after 12 weeks of Provox XtraHME (second generation) use (p<0.001) (a lower score means les burden to the patient) on the Structured Questionnaires [42].

9A. Please enter weights in the table below which indicate what percentage of patients you expect to experience psychosocial problems after 12 weeks of Provox Life use.

Enter your weights of belief in each of the possible intervals of percentage shown in the table below. The stronger you believe that the percentage of patients experiencing psychosocial problems will truly lie in a given interval, the greater should be your weight for that interval (maximum weight of 100). If you believe the percentage of experiencing psychosocial problems does not lie in a given interval, your weight should be zero for that specific interval. Please insert an answer in every row.

9B. Please add up your weight in the final column of the table below. These should add up to 100.

Please fill in your answers in this table (use your mouse to go to the next box)**:**

After 12 weeks of Provox Life use, ..% of patients will **experience psychosocial problems:**

% Patients	0-10	11-20	21-30	31-40	41-50	51-60	61-70	71-80	81-90	91-100	**TOTAL** (should add up to 100)
Weight											

10. Physiotherapy

The use of Provox XtraHME (second generation) statistically significant reduced the average number of days requiring chest physiotherapy post-surgery (1.75 days vs. 3.20 days, p = 0.034) compared to external humidifier (EH) users [44].

10A. Please enter weights in the two tables below which indicate what increase (table 1) or decrease (table 2) in average number of days you expect patients require chest physiotherapy post-surgery after 12 weeks of Provox Life use compared to patients using Provox XtraHME (second generation).

Enter your weights of belief in each of the possible intervals of percentage shown in the table below. The stronger you believe that the difference in the average number of days required post-surgery physiotherapy will truly lie in a given interval, the greater should be your weight for that interval (maximum weight of 100). If you believe the difference in the average number of days requiring post-surgery physiotherapy does not lie in a given interval, your weight should be zero for that specific interval. Please insert an answer in every row.

10B. Please sum your weights across both tables in the final row of the second table below. The total sum should be 100.

Please fill in your answers in the tables below (use your mouse to go to the next box):

Table 1:

Increase in the average number of days requiring chest physiotherapy in patients using Provox Life compared to Provox XtraHME (second generation) use after 12 weeks:

Average number of days	>6,0	6.0-5.1	5.0-4.1	4.0-3.1	3.0-2.1	2.0-1.1	1.0-0	TOTAL (should add up to 100)
Weight								

Table 2:

Decrease in the average **number of days requiring chest physiotherapy** in patients using Provox Life compared to Provox XtraHME (second generation) use after 12 weeks:

Average number of days	0-1,0	1,1-2,0	2,1-3,0	3,1-4,0	4,1-5,0	5,1-6,0	>6,0	**TOTAL** (should add up to 100)
Weight								

11.Tracheobronchitis and/or pneumonia episodes

Tracheobronchitis occurs when the windpipe or bronchi become inflamed. This is usually due to a viral or bacterial infection, but it can also be the result of some kind of irritant. It is rare for people with acute tracheobronchitis to develop complications. However, in a very small number of cases, people may go on to develop pneumonia (an infection in one or both lungs) [39].

A clinical study observed 0.285 tracheobronchitis and/or pneumonia episodes per patient/year in non-HME users, which was statistically significant higher than the 0.066 episodes per patient/year in HME users (first generation) (p=0.047). Besides, in non-HME users, an average of 0.129 pulmonary infections (tracheobronchitis and pneumonia together) per patient/year was documented. For the HME users (first generation) this average was 0.092 per patient/year (p=0.33) [12].

11A. Please enter weights in the two tables below which indicate what percentage decrease (table 1) or increase (table 2) in pulmonary infections (tracheobronchitis and pneumonia together) you expect on a yearly basis per patient when comparing Provox Life use to Provox XtraHME use.

Enter your weights of belief in each of the possible intervals of percentage shown in the table below. The stronger you believe that the percentage increase or decrease in pulmonary infections will truly lie in a given interval, the greater should be your weight for that interval (maximum weight of 100). If you believe the percentage of increase or decrease does not lie in a given interval, your weight should be zero for that specific interval. Please insert an answer in every row.

11B. Please sum your weights across both tables in the final row of the second table below. The total sum should be 100.

Please fill in your answers in the tables below (use your mouse to go to the next box):

Table 1:

Percentage of **decrease in pulmonary infections** in patients using Provox Life compared to Provox XtraHME (second generation) use:

% Decrease in pulmo-nary infections	100-91	90-80	80-71	70-61	60-51	50-40	40-31	30-21	20-11	10-0
Weight										

Table 2:

Percentage of **increase in pulmonary infections** in patients using Provox Life compared to Provox XtraHME (second generation) use:

% Increase in pulmo-nary infections	0-10	11-20	21-30	31-40	41-50	51-60	61-70	71-80	81-90	91-100	**TOTAL** (should add up to 100)
Weight											

12.Social contacts

Non-HME users, Provox Micron HME (first generation) users, and Provox HME (first generation) users scored a 9.6, 8.4 and 9.7 respectively on the frequency of social contacts category from the Quality-of-Life Questionnaire (p=0.438) (a higher score indicates more problems) [16].

Another study however observed no statistically significant change (p=0.728) in social contacts when comparing no HME (second generation) use to 2, 6 or 12 weeks of HME use [42].

12A. Please enter weights in the two tables below which indicate what percentage decrease (table 1) or increase (table 2) in average number of social contacts you expect for patients after 12 weeks of Provox Life use compared to patients using Provox XtraHME.

Enter your weights of belief in each of the possible intervals of percentage shown in the table below. The stronger you believe that the percentage increase or decrease in average number of social contacts will truly lie in a given interval, the greater should be your weight for that interval (maximum weight of 100). If you believe the percentage of increase or decrease does not lie in a given interval, your weight should be zero for that specific interval. Please insert an answer in every row.

12B. Please sum your weights across both tables in the final row of the second table below. The total sum should be 100.

Please fill in your answers in the tables below (use your mouse to go to the next box):

Table 1:

Percentage of **decrease in average number of social contacts** in patients using Provox Life compared to Provox XtraHME (second generation) use after 12 weeks:

% Decrease in average number of social contacts	100-91	90-80	80-71	70-61	60-51	50-40	40-31	30-21	20-11	10-0
Weight										

Table 2:

Percentage of **increase in average number of social contacts** in patients using Provox Life compared to Provox XtraHME (second generation) use after 12 weeks:

% Increase in average number of social contacts	0-10	11-20	21-30	31-40	41-50	51-60	61-70	71-80	81-90	91-100	**TOTAL** (should add up to 100)
Weight											

13.Quality of life

Many total laryngectomy patients suffer from both physical and psychosocial postoperative problems, which can have a major impact on their quality of life (QoL). QoL among total laryngectomy patients is mainly influenced by issues related to limitations in daily activities and avoidance of social activities and underlying pulmonary problems[7].

Literature shows that the use of HME can positively influence QoL of total laryngectomy patients;

- an increase from 61.3 at baseline to 80.0 after 12 weeks of Provox XtraHME (second generation) use ($p < .0001$) was found when using the EQ-5D VAS scale [42].
- another study including 60 patients, who were randomised between a control group that used no device and a group equipped with the Provox HME (first generation), reported that 92% of the patients with the device perceived an improvement in their QoL (no p-value reported) [40].

13A. Please enter weights in the two tables below which indicate what percentage of decrease (table 1) or increase (table 2) in QoL you expect after 12 weeks of Provox Life use compared to Provox XtraHME (second generation) use.

Enter your weights of belief in each of the possible intervals of percentage shown in the table below. The stronger you believe that the percentage increase or decrease in QoL will truly lie in a given interval, the greater should be your weight for that interval (maximum weight of 100). If you believe the percentage of increase or decrease does not lie in a given interval, your weight should be zero for that specific interval. Please insert an answer in every row.

13B. Please sum your weights across both tables in the final row of the second table below. The total sum should be 100.

Please fill in your answers in the tables below (use your mouse to go to the next box):

[7] Atos Medical, unpublished data, 2021

Table 1:

Percentage of **decrease in overall QoL** in patients using Provox Life compared to Provox XtraHME (second generation) use after 12 weeks:

% Decrease in QoL	100-91	90-80	80-71	70-61	60-51	50-40	40-31	30-21	20-11	10-0
Weight										

Table 2:

Percentage of **increase in overall QoL** in patients using Provox Life compared to Provox XtraHME (second generation) use after 12 weeks:

% Increase in QoL	0-10	11-20	21-30	31-40	41-50	51-60	61-70	71-80	81-90	91-100	**TOTAL** (should add up to 100)
Weight											

14.Patient satisfaction

Patients' satisfaction is significantly higher for first generation HME use verses external humidifier (EH) use (p<.001). Of patients in the EH group, 11% reported that they were satisfied with EH, 8% reported they liked it somewhat, and 81% reported that they did not like the EH. Of the patients in the HME (first generation) group 100% were satisfied [29].

After 12 weeks of use, 60.7% of the patients were 'very satisfied' with the use of the HME (first generation), and 39.3% were 'satisfied'. None of the patients was dissatisfied with the XtraHME (second generation) [41].

14A. Please enter weights in the two tables below which indicate what percentage of patients you expect to experience either a decrease (table 1) or an increase (table 2) in satisfaction when comparing Provox Life with Provox XtraHME (second generation) after 12 weeks.

Enter your weights of belief in each of the possible intervals of percentage shown in the table below. The stronger you believe that the percentage of patient experiencing an increase or decrease in patient satisfaction will truly lie in a given interval, the greater should be your weight for that interval (maximum weight of 100). If you believe the percentage of increase or decrease does not lie in a given interval, your weight should be zero for that specific interval. Please insert an answer in every row.

14B. Please sum your weights across both tables in the final row of the second table below. The total sum should be 100.

Please fill in your answers in the tables below (use your mouse to go to the next box)**:**

Table 1:

Use of Provox Life results in ..% of patients being **less satisfied** about their HME when compared to patients using Provox XtraHME (second generation) after 12 weeks:

% Patients	100-91	90-80	80-71	70-61	60-51	50-40	40-31	30-21	20-11	10-0
Weight										

Table 2:

Use of Provox Life results in ..% of patients being **more satisfied** about their HME when compared to patients using Provox XtraHME (second generation) after 12 weeks:

% Patients	0-10	11-20	21-30	31-40	41-50	51-60	61-70	71-80	81-90	91-100	**TOTAL** (should add up to 100)
Weight											

You have reached THE END of this survey.

Thank you very much for your answers.

If you have questions and/or remarks,

please contact [email address] or use the textbox below

____
