# Supplementary material for: New Heat and Moisture Exchangers for Laryngectomized Patients in Germany: Mixed Methods Study on the Expected Effectiveness
Source: JMIR Form Res. 2023 Jan 11;7:e36401. doi: 10.2196/36401 (PMC9878367; doi:10.2196/36401)
Supplement: Multimedia Appendix 4 [file formative_v7i1e36401_app4.pdf]

Multimedia Appendix 4 – Average scores of expert judgements on effect variables

Variable	Interpretation		Average	SD	n
Breathing	Use of new HMEs results in 53 % of patients breathing better	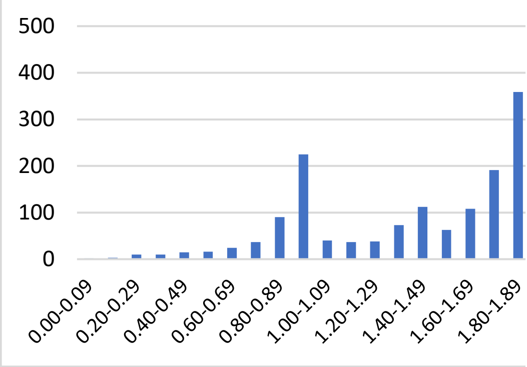<br><br>0-1 indicating breathing worse, 1-2 indicating breathing better	1,53	0,28	19
Shortness of breath	48% of patients will experience a decrease in shortness of breath when using the new HMEs	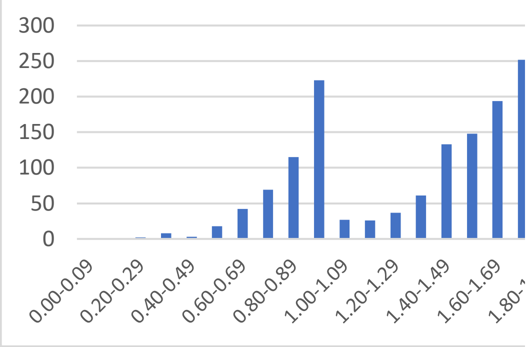<br><br>0-1 indicating breathing worse, 1-2 indicating breathing better	1,48	0,25	19
Tracheal Climate	59% of patients experience a decrease in tracheal dryness/ irritation when using the new HMEs	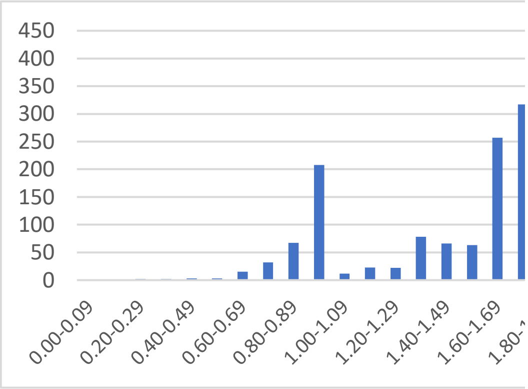<br><br>0-1 indicating an increase in tracheal dryness/irritation, 1-2 indicating a decrease in tracheal dryness/irritation	1,59	0,19	19

	51% of patients will experience a decrease in tracheal dryness/irritation when using the new HMEs compared to second generation HMEs	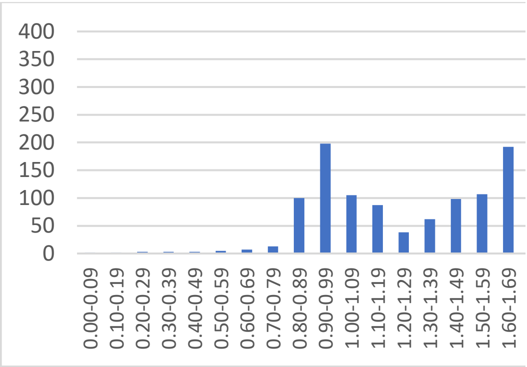<br>0-1 indicating an increase in tracheal dryness/irritation, 1-2 indicating a decrease in tracheal dryness/irritation	1,51	0,24	19
Mucus production /plugging	Percentage of decrease in patients experiencing mucus plug events when using new HMEs compared to patients using second generation HMEs after the acute postoperative period (2 weeks) is 33%	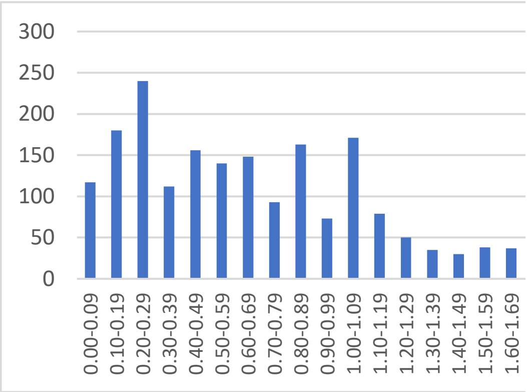<br>0-1 indicating percentage of decrease in patients experiencing mucus plug events, 1-2 indicating percentage of increase in patients experiencing mucus plug events	0,67	0,32	19
	After 12 weeks of using the new HMEs, 53% of patients will have decreased mucus production compared to no HME use	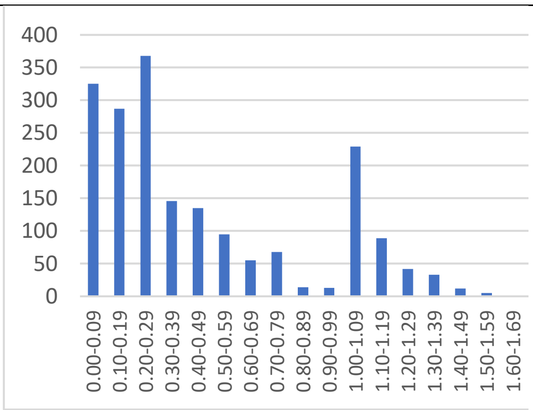<br>0-1 indicating percentage of patients with decreased mucus production, 1-2 indicating percentage of patients with increased mucus production	0,47	0,22	19

Coughing	Average amount of daily coughs per patients after 12 weeks of using the new HMEs is 2.95	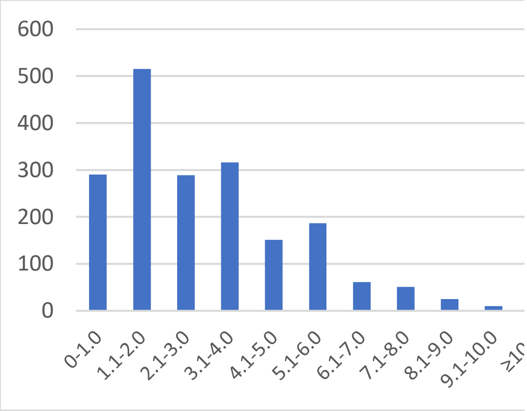<br>X-Axis displaying the amount of daily coughs per patient	2.95	1.61	19
Forced Expectorati ons	Average number of daily forced expectorations per patients after 12 weeks of new HME use is 2.46	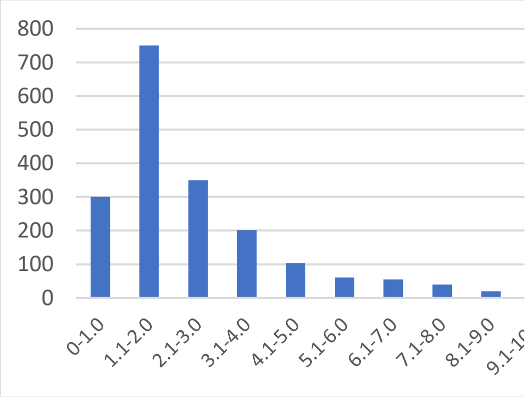<br>X-Axis displaying the amount of daily forced expectorations per patient	2.46	1.42	19
Sleep quality	48% of patients will experience sleeping problems after 12 weeks of using the new HMEs	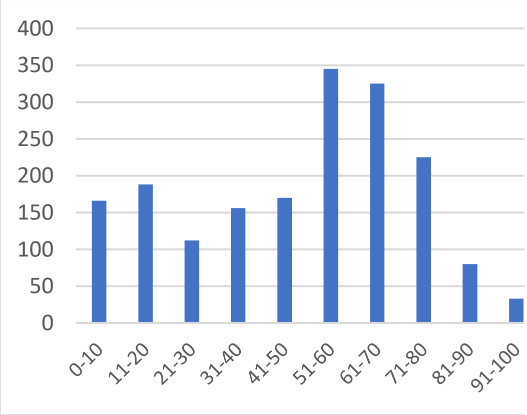<br>X-axis indicating percentage of patients experiencing sleeping problems	0,48	0,22	18

Speech quality	12 weeks use of the new HMEs results in 25% of patients with better speech quality compared to second generation HME use	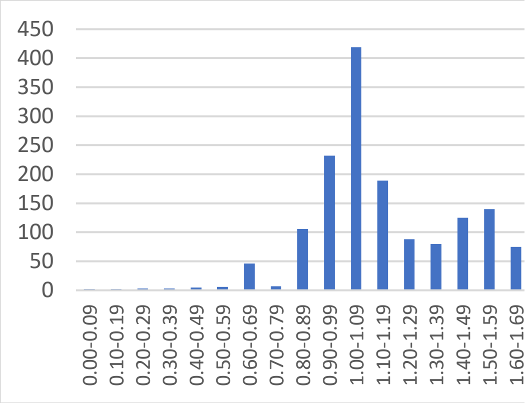<br>0-1 indicating patients with worse speech quality, 1-2 indicating patients with better speech quality	1,25	0,23	18
Psycho-social aspects	After 12 weeks of using new HMEs 24% of patients will experience psychosocial problems	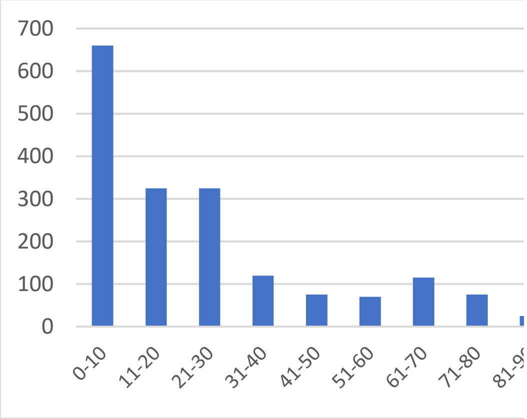<br>X-axis indicating percentage of patients experiencing psychosocial problems	0,24	0,20	18
Physio-therapy	Decrease in the average number of days requiring chest physiotherapy in patients using new HMEs compared to second generation HME use after 12 weeks is 0.74 days	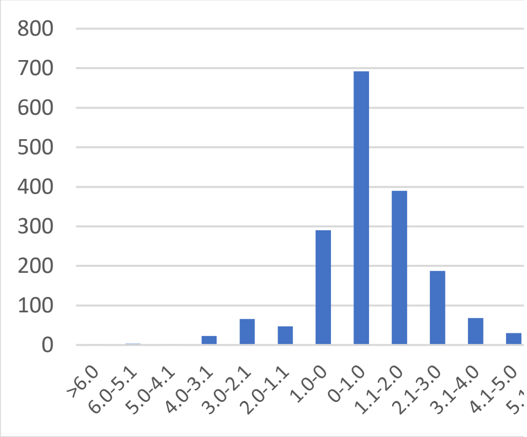<br>>6-0 indicating an increase in the average number of days requiring chest physiotherapy,0->6 indicating a decrease in the average number of days requiring chest physiotherapy	0.74	0.70	18

<table>
<tr><th></th><th></th><th></th><th></th><th></th><th></th></tr>
<tr>
<td>*Tracheo-bronchitis and/or pneumonia episodes*</td>
<td>Percentage of decrease in pulmonary infections in patients new HMEs compared to second generation HME use is 34%</td>
<td>
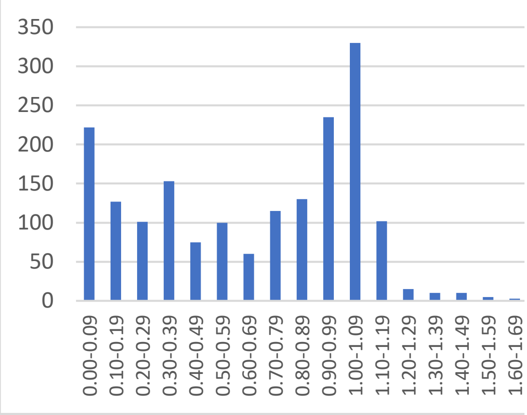

0-1 indicating decrease in pulmonary infections, 1-2 indicating increase in pulmonary infection
</td>
<td>0,66</td>
<td>0,32</td>
<td>18</td>
</tr>
<tr>
<td>*Social contacts*</td>
<td>Percentage of increase in average number of social contacts in patients using new HMEs compared to second generation HME use after 12 weeks is 13%</td>
<td>
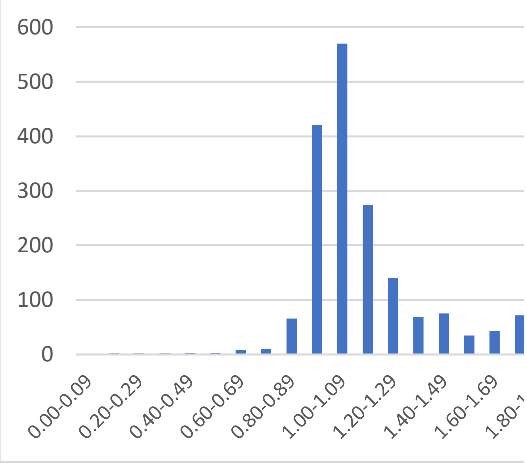

0-1 indicating percentage of decrease in average number of social contacts, 1-2 indicating percentage of increase in average number of social contacts
</td>
<td>1,13</td>
<td>0,18</td>
<td>18</td>
</tr>
<tr>
<td>*Quality of Life*</td>
<td>Percentage of increase in overall QoL in patients using new HMEs compared to second generation use after 12 weeks is 33%</td>
<td>
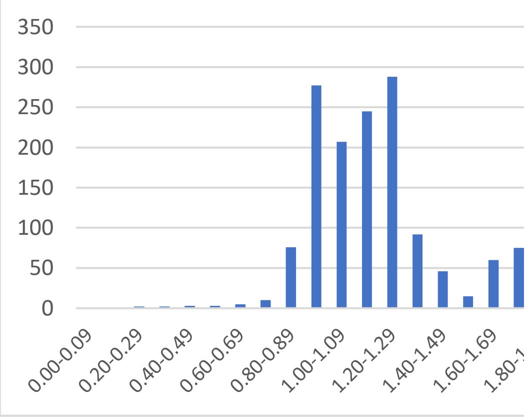

0-1 indicating percentage decrease in overall QoL, 1-2 indicating percentage increase in overall QoL
</td>
<td>1,33</td>
<td>0,30</td>
<td>18</td>
</tr>
</table>

Patient satisfaction	Use of new HMEs results in 44% of patients being more satisfied about their HME when compared to patients using second generation HMEs after 12 weeks	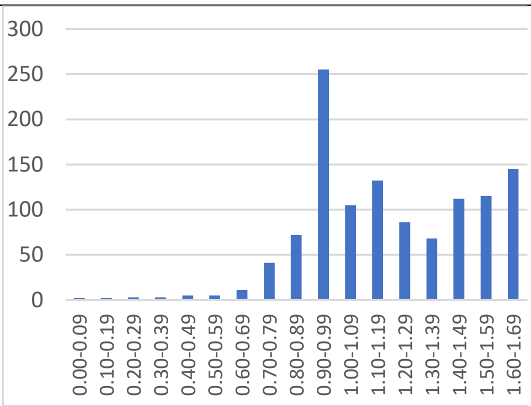<br>0-1 indicating percentage of patients being less satisfied, 1-2 indicating percentage of patients being more satisfied	1,44	0,30	18

n: number of experts; SD: standard deviation; HMEs: Heat and Moisture Exhangers; QoL: Quality of Life
